# Supplementary material for: Year-round temporal stability of a tropical, urban plant-pollinator network
Source: PLoS One. 2020 Apr 10;15(4):e0230490. doi: 10.1371/journal.pone.0230490 (PMC7147774; doi:10.1371/journal.pone.0230490)
Supplement: S1 Fig — (PDF) [file pone.0230490.s001.pdf]

S1 Figure. Map of study parks in Bangkok, Thailand.

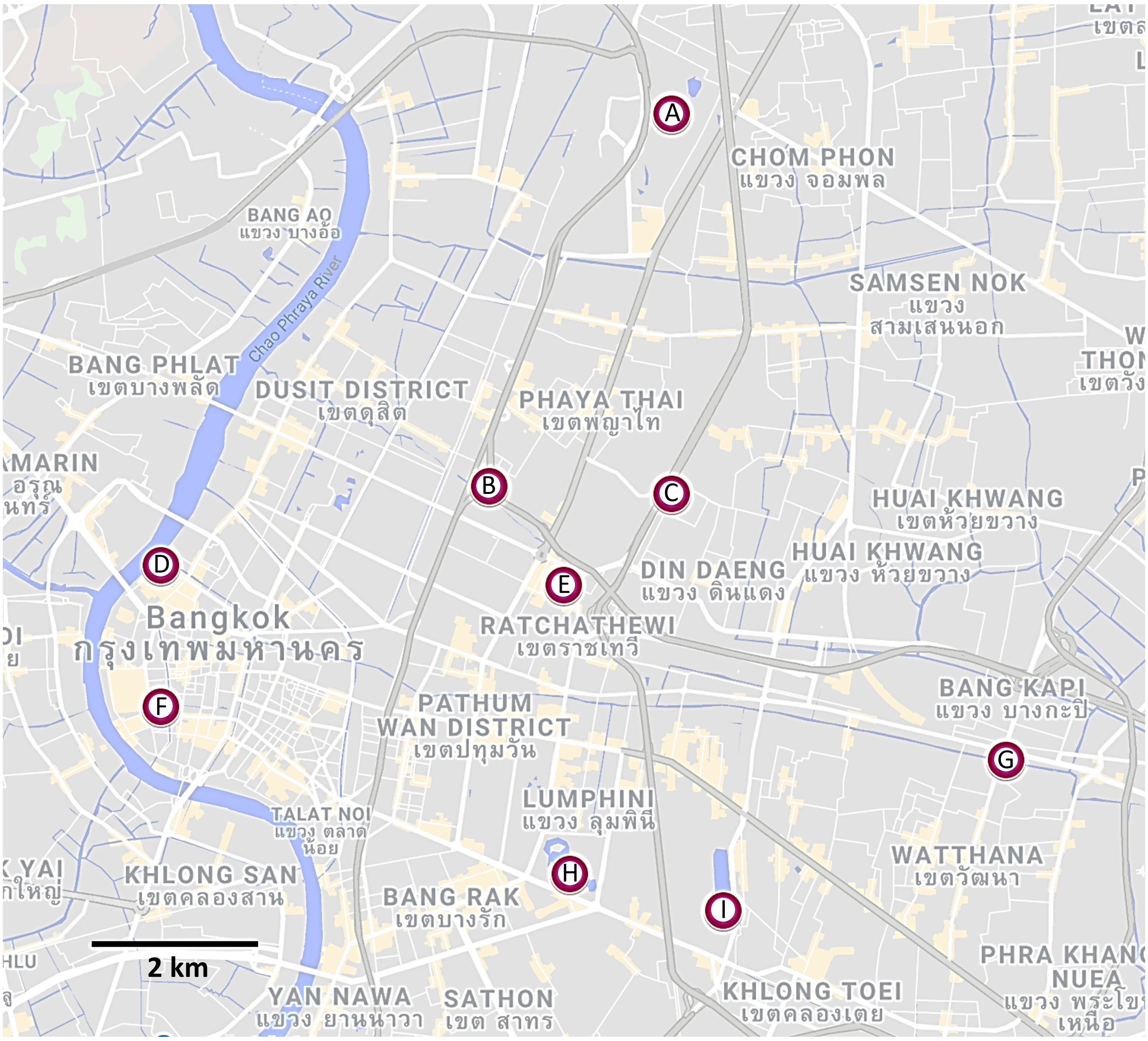

(A) Wachirabenchathat Park (1,097,600 m^2). (B) Phaya Thai Pirom Garden (28,600 m^2). (C) Vibhavadi Rangsit Forest Park (23,300 m^2). (D) Santi Chai Prakan Public Park (13,600 m^2). (E) Santiphap Park (32,000 m^2). (F) Saranrom Park (36,800 m^2). (G) Somdet Saranrat Maneerom Public Park (34,500 m^2). (H) Lumphini Park (576,000 m^2). (I) Benjakitti Park (108,000 m^2).
