# Supplementary material for: Year-round temporal stability of a tropical, urban plant-pollinator network
Source: PLoS One. 2020 Apr 10;15(4):e0230490. doi: 10.1371/journal.pone.0230490 (PMC7147774; doi:10.1371/journal.pone.0230490)

S2 Figure. Plant-pollinator networks at each study park in Bangkok, Thailand over 12 months.

(A) Total plant-pollinator network using data from all 9 parks over all 12 months. Individual plant-pollinator networks from each study month (December 2017 – November 2018) at (B) Benjakitti Park, (C) Lumphini Park, (D) Phaya Thai Pirom Garden, (E) Santi Chai Prakan Public Park, (F) Santiphap Park, (G) Saranrom Park, (H) Somdet Saranrat Maneerom Public Park, (I) Vibhavadi Rangsit Forest Park, and (J) Wachirabenchathat Park. Upper bars represent pollinator species, lower bars represent plant species, and connecting lines represent interactions; widths are proportional to the number of individuals or the number of times that an interaction occurred. Pollinator species names can be found in S1 Table and plant species names can be found in S2 Table. Months in which no pollination interactions were observed are denoted as "N/A".

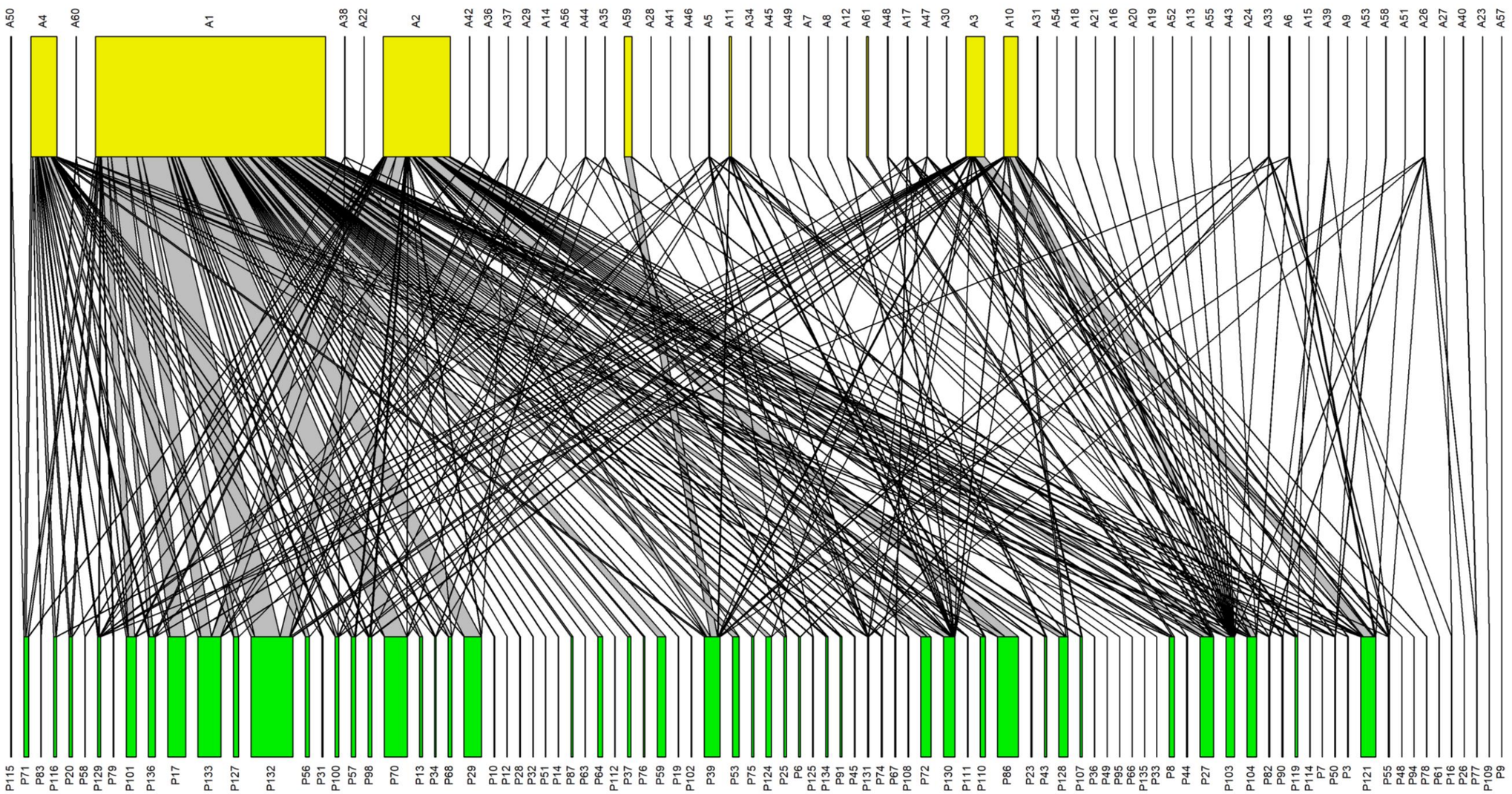

(B) Benjakitti Park

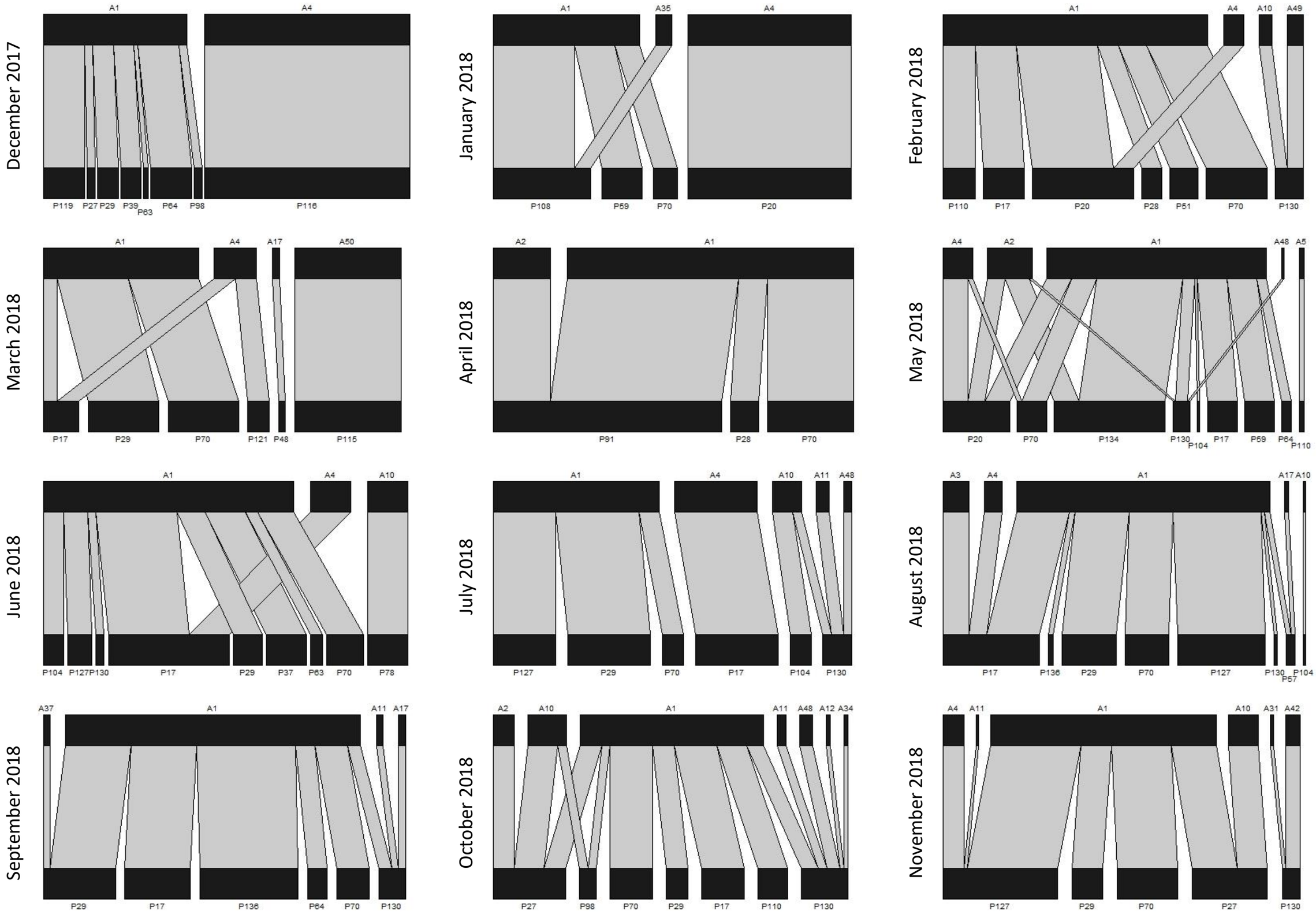

(C) Lumphini Park

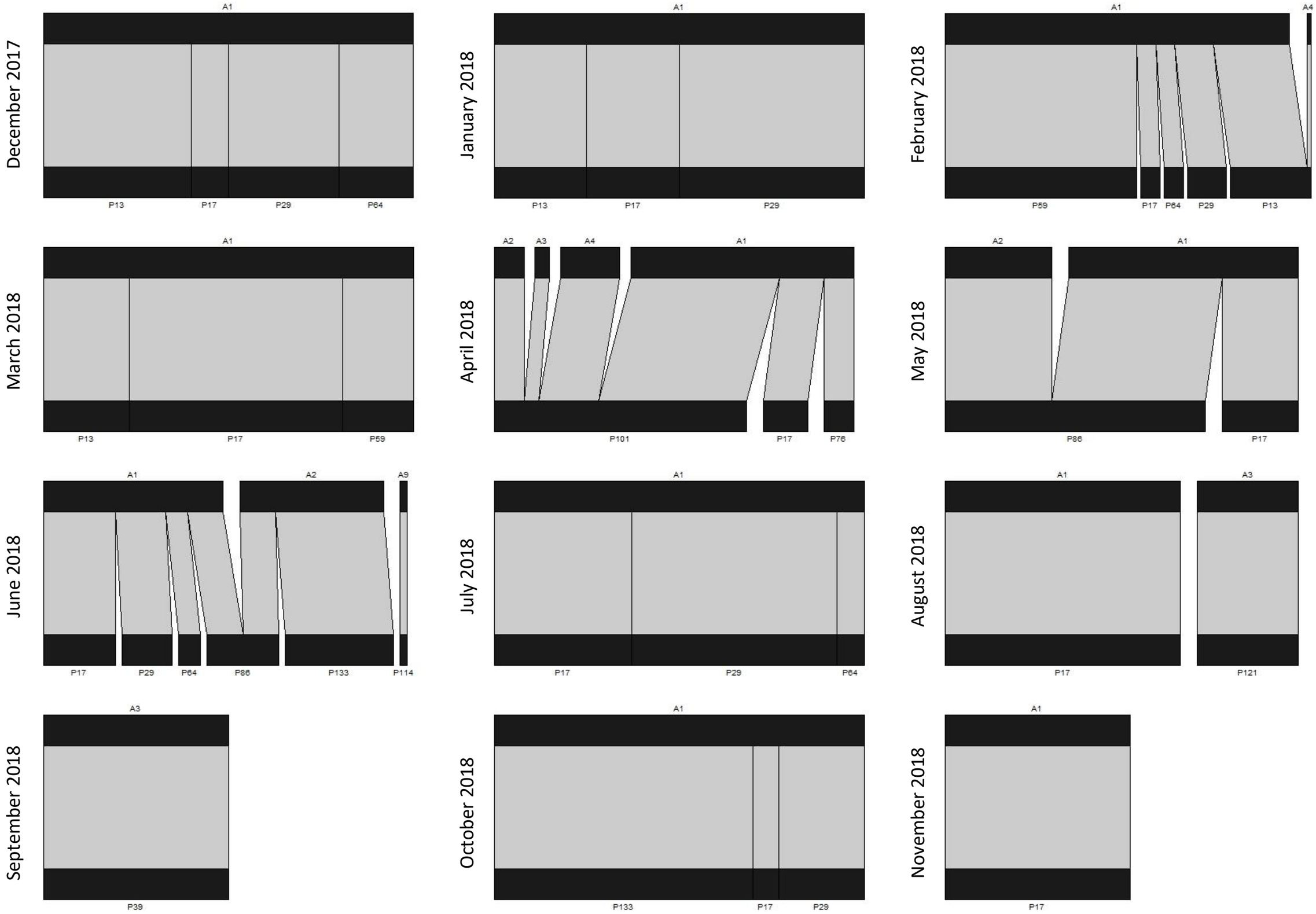

(D) Phaya Thai Pirom Garden

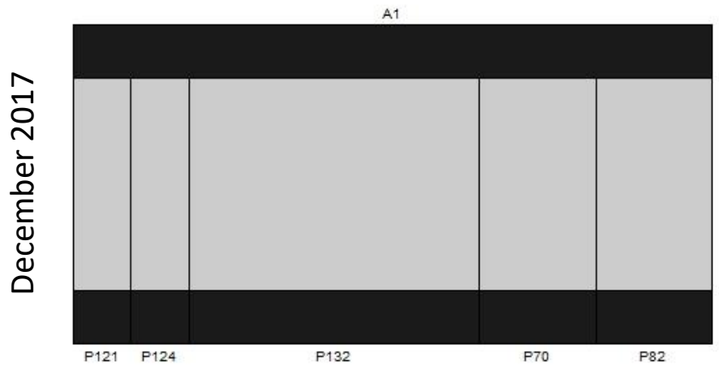

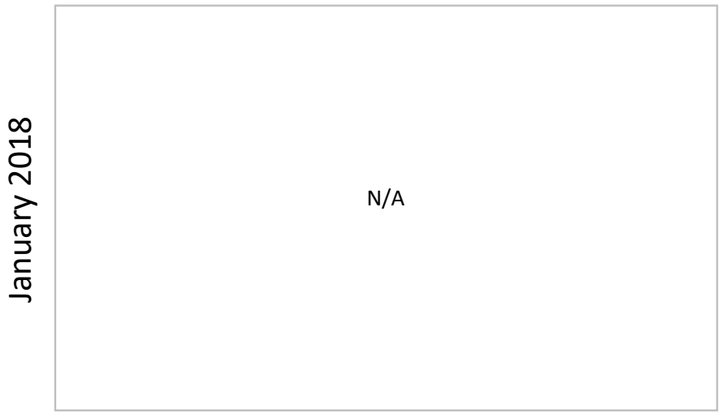

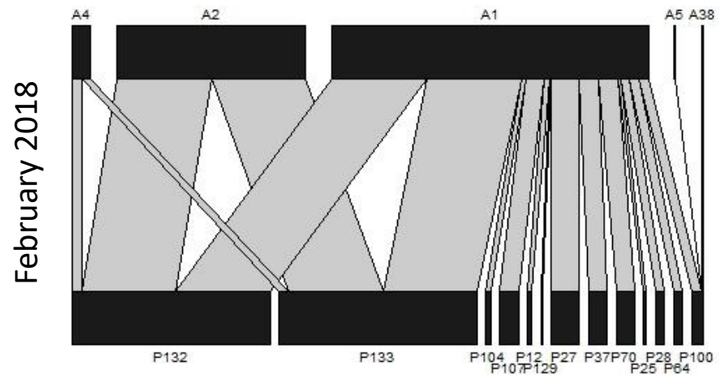

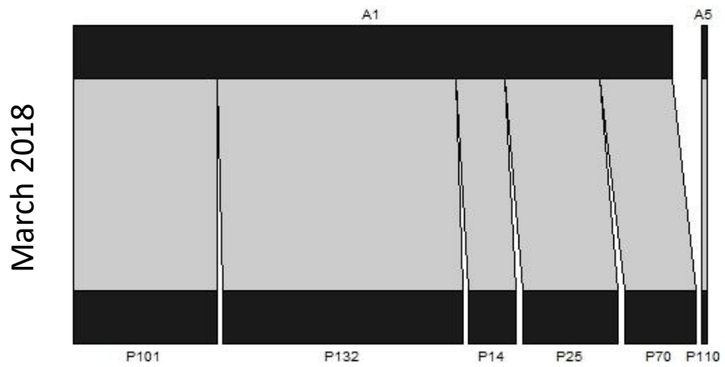

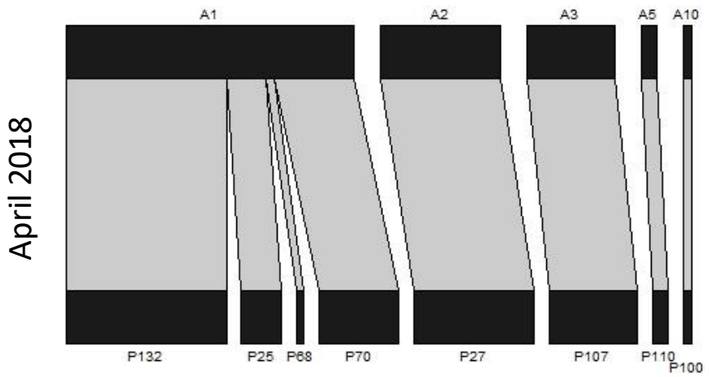

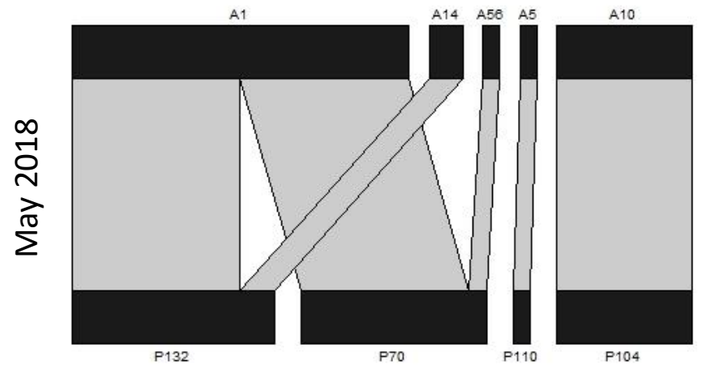

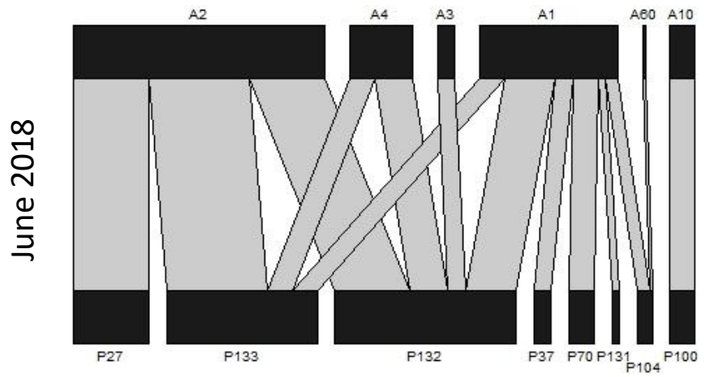

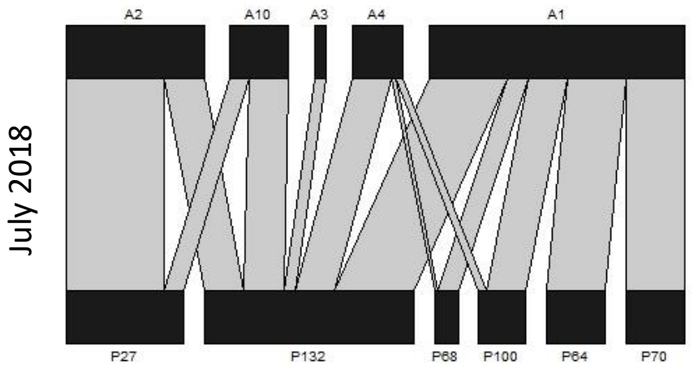

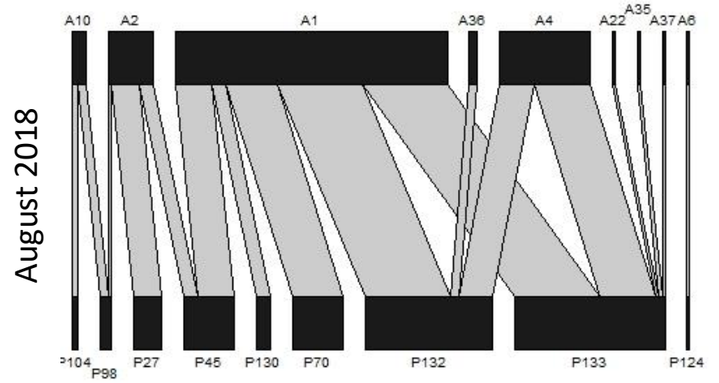

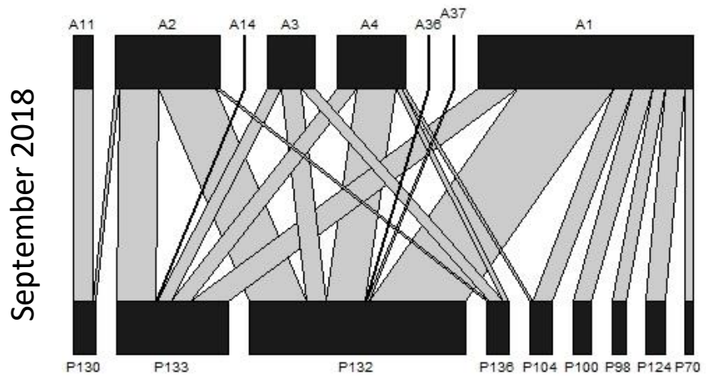

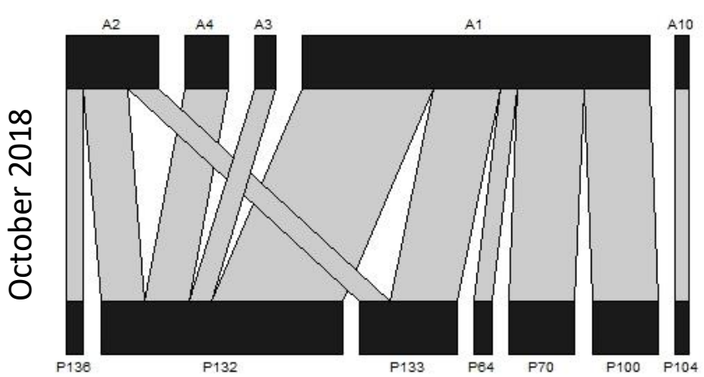

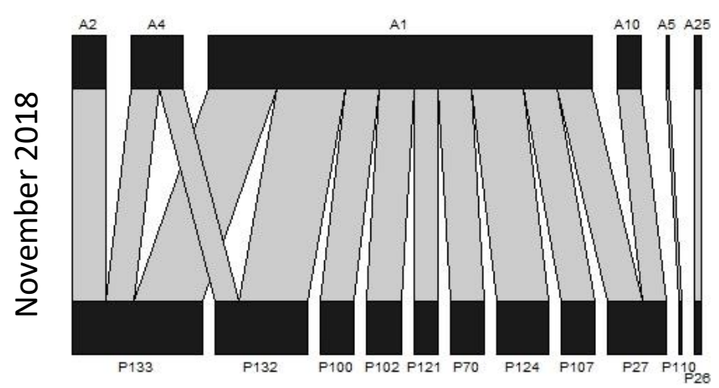

(E) Santi Chai Prakan Public Park

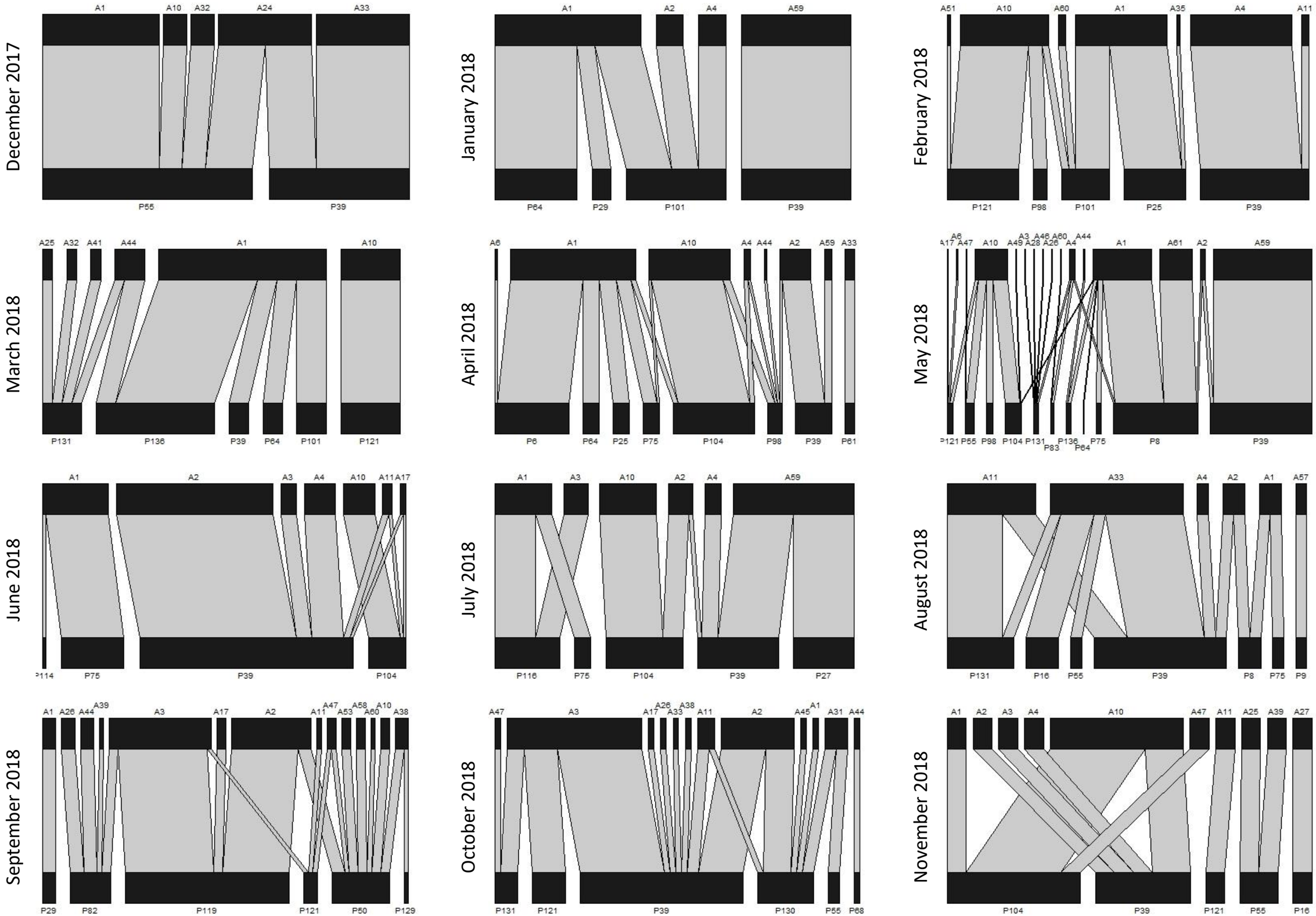

(F) Santiphap Park

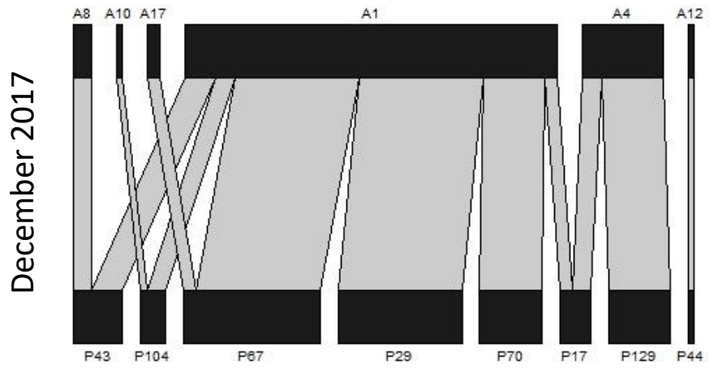

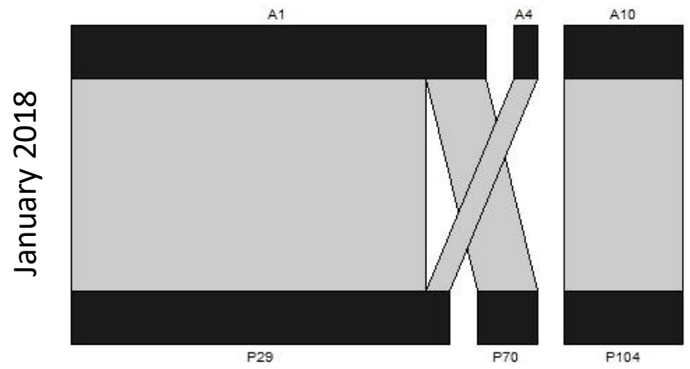

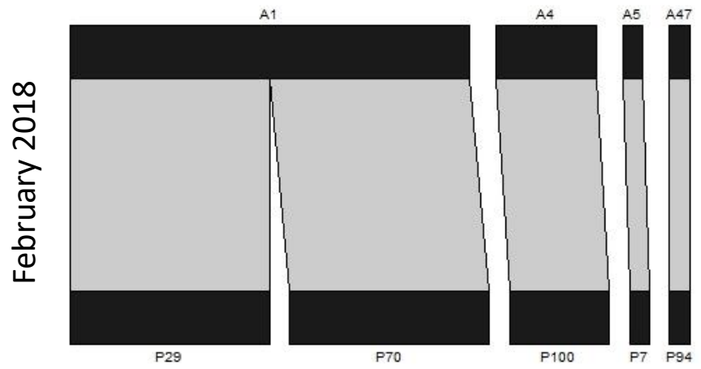

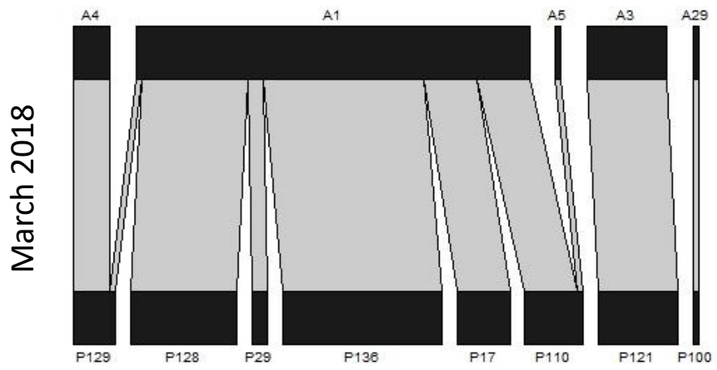

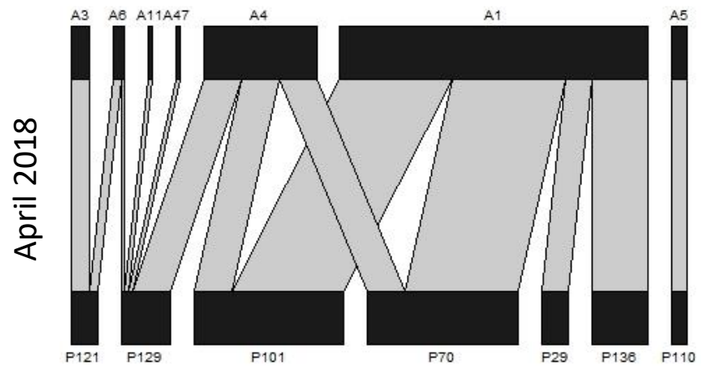

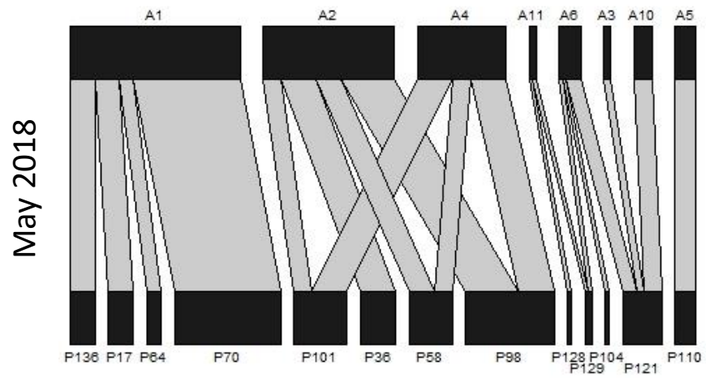

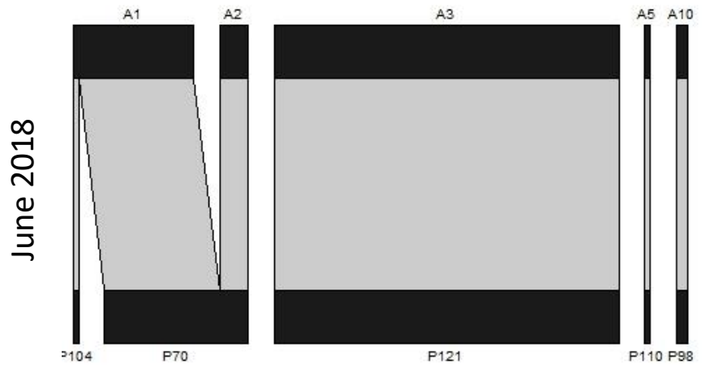

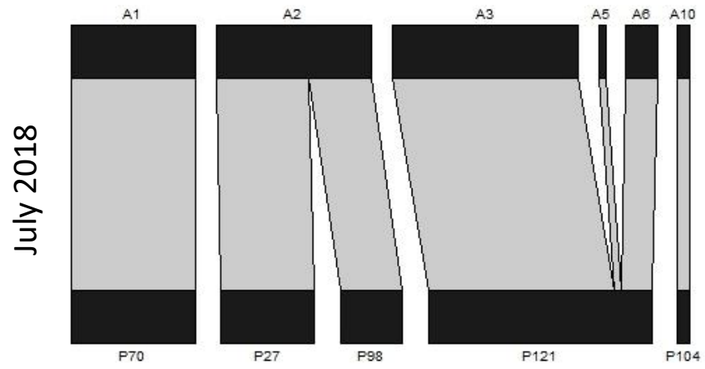

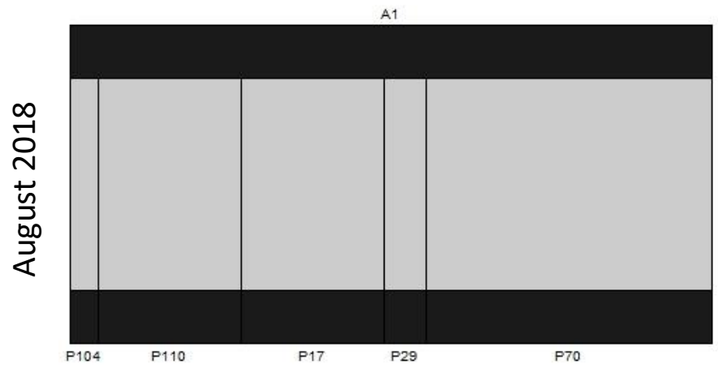

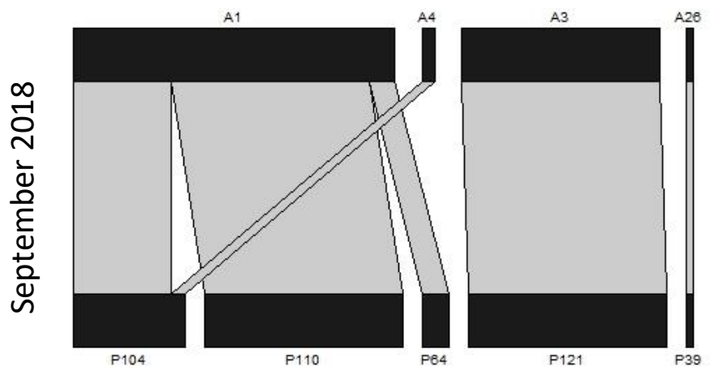

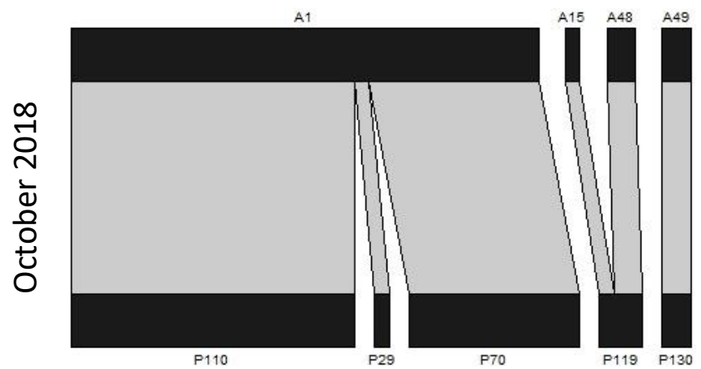

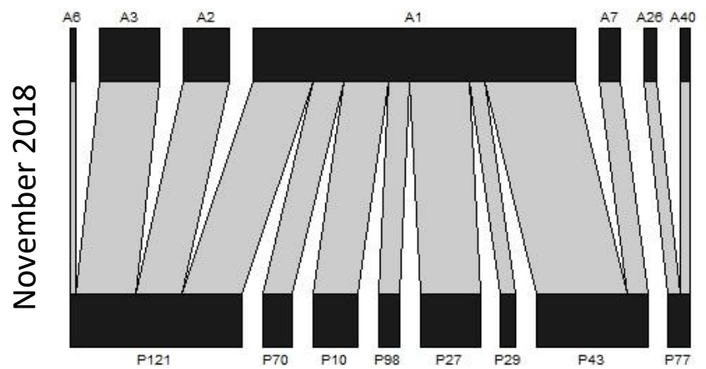

(G) Saranrom Park

December 2017

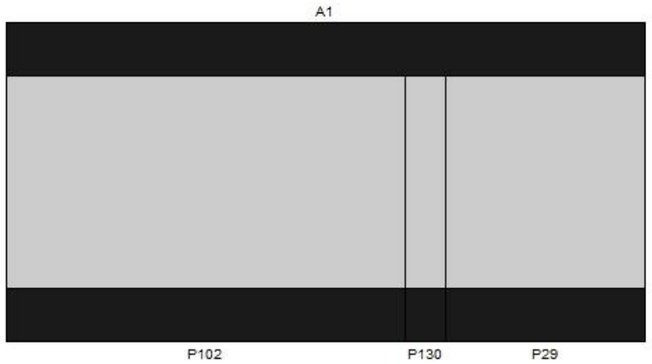

January 2018

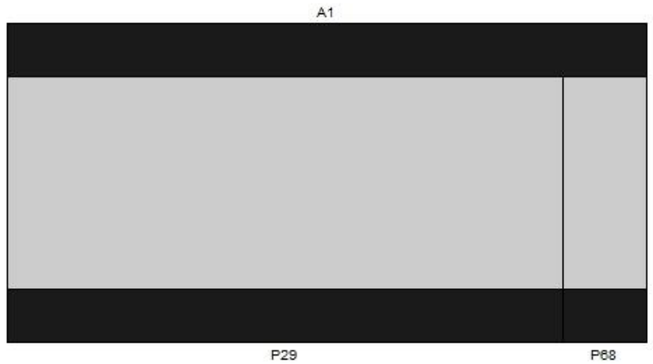

February 2018

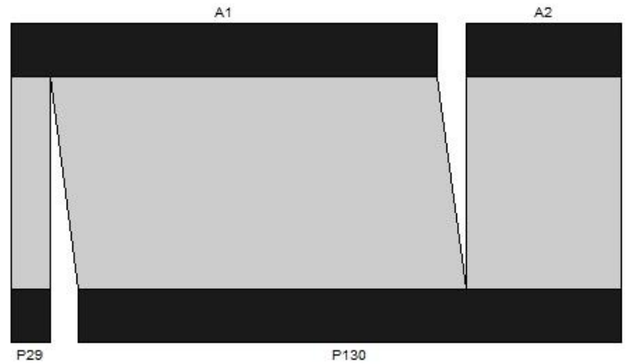

March 2018

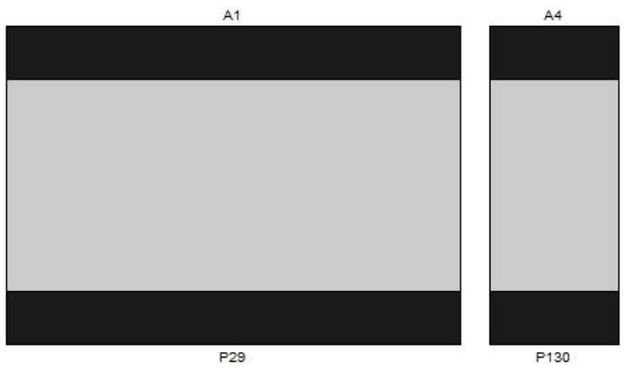

April 2018

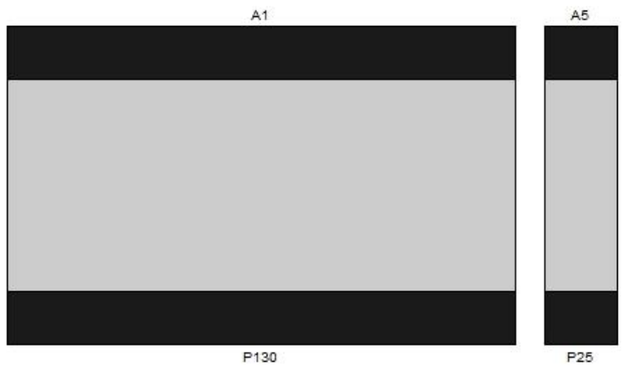

May 2018

N/A

June 2018

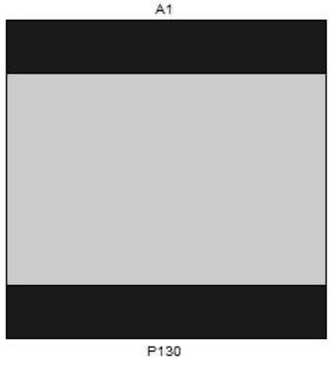

July 2018

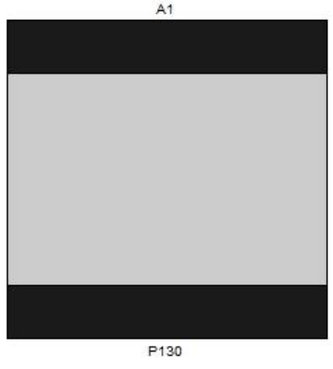

August 2018

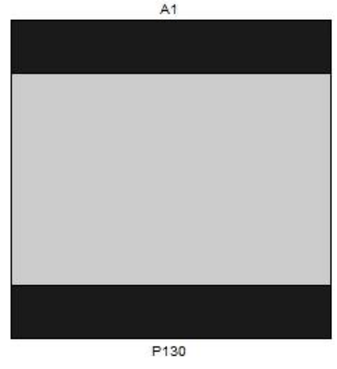

September 2018

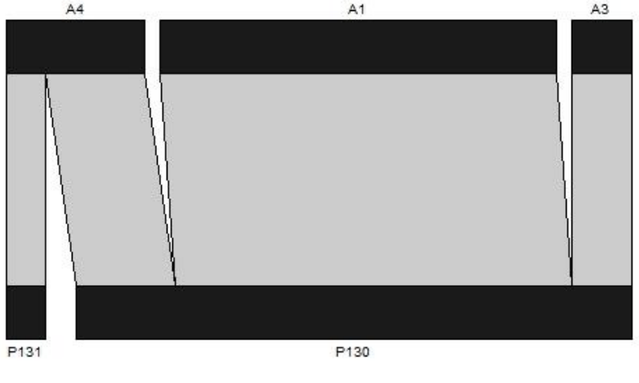

October 2018

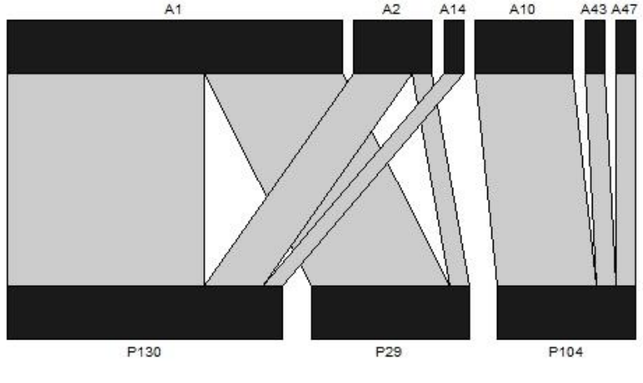

November 2018

N/A

(H) Somdet Saranrat Maneerom Public

December 2017

N/A

January 2018

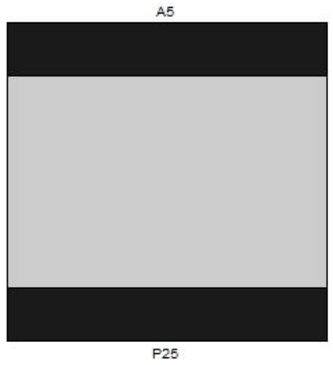

February 2018

N/A

March 2018

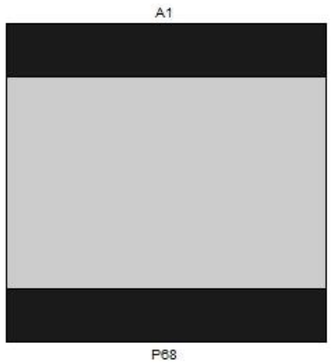

April 2018

N/A

May 2018

N/A

June 2018

N/A

July 2018

N/A

August 2018

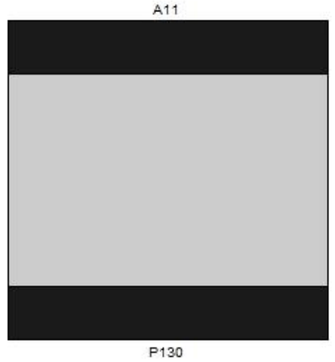

September 2018

N/A

October 2018

N/A

November 2018

N/A

(I) Vibhavadi Rangsit Forest Park

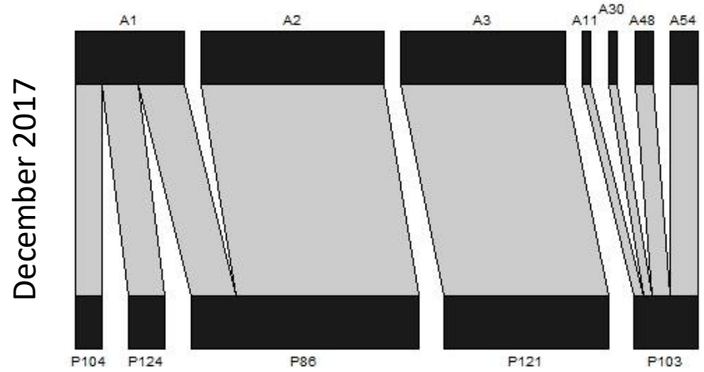

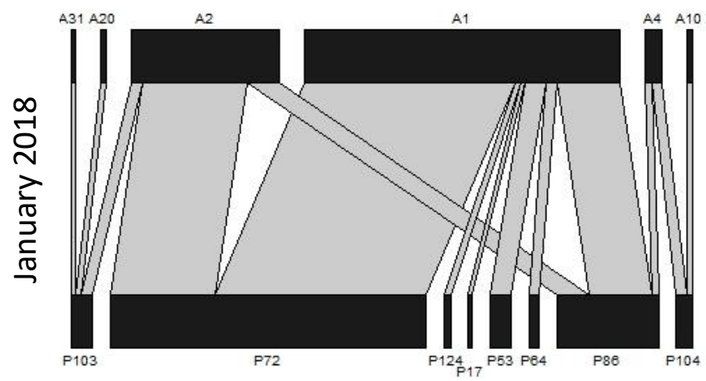

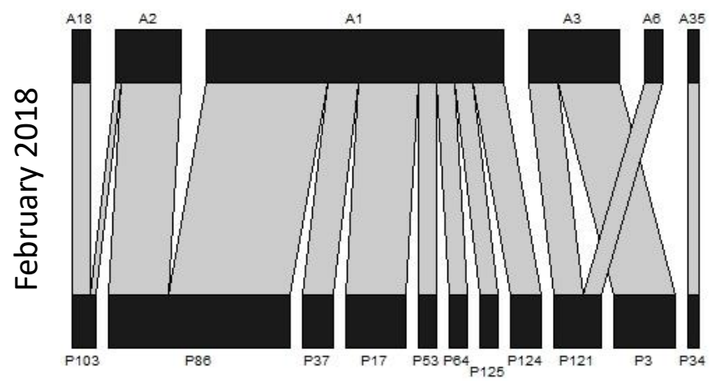

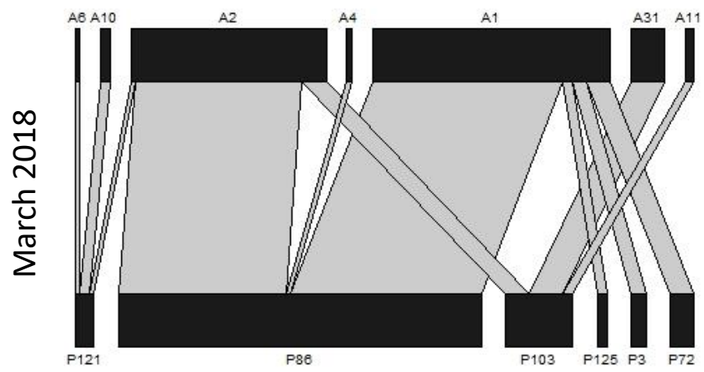

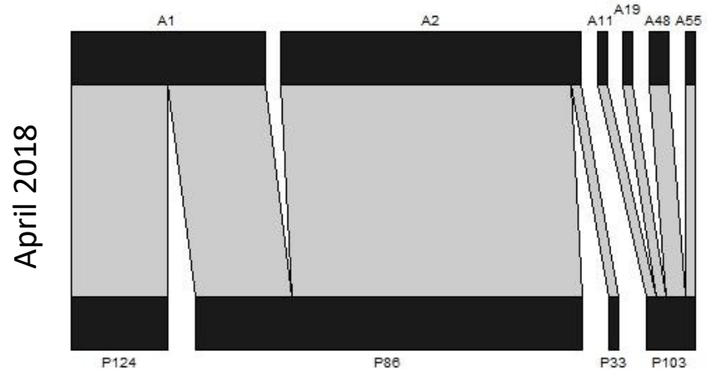

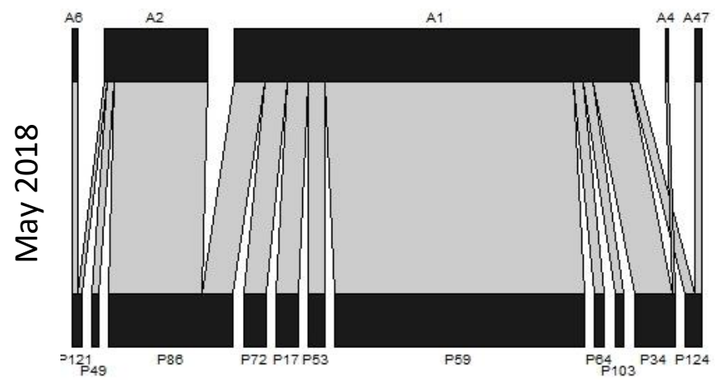

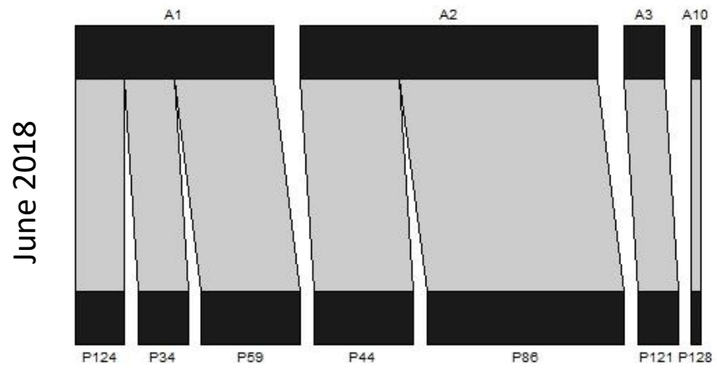

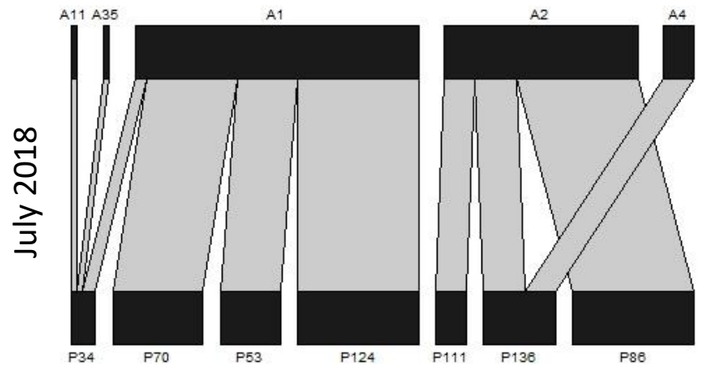

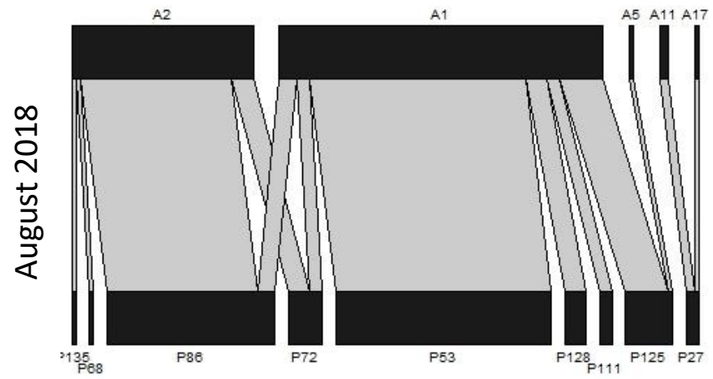

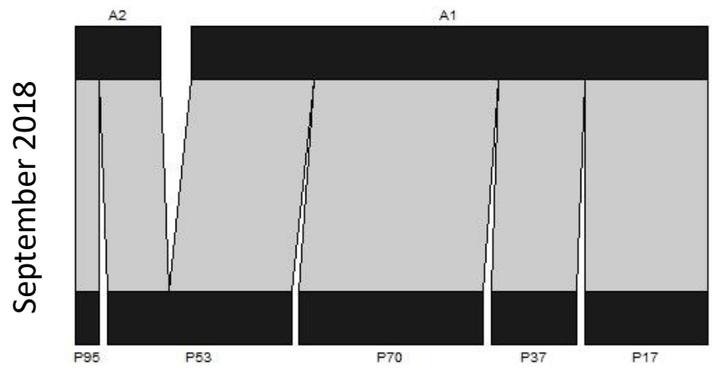

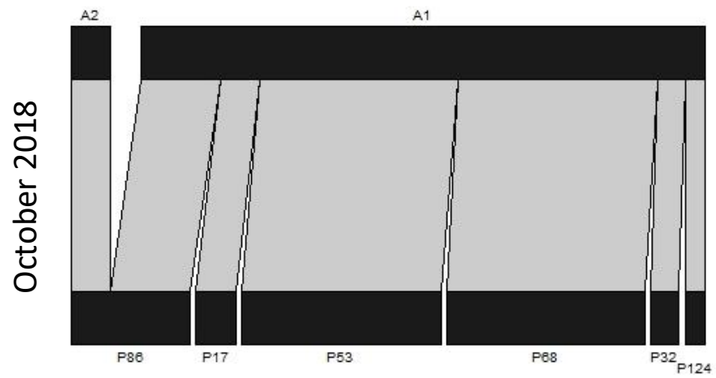

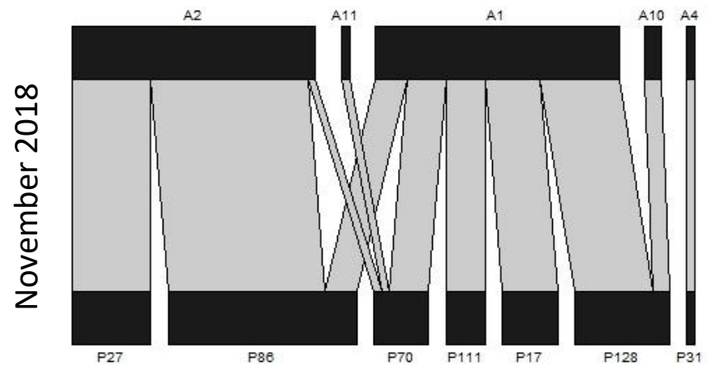

(J) Wachirabenchathat Park

December 2017

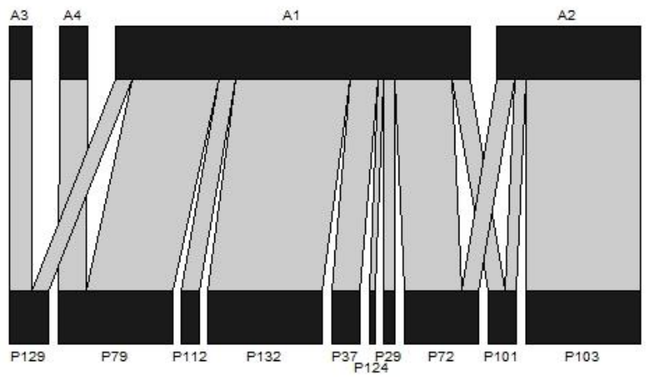

January 2018

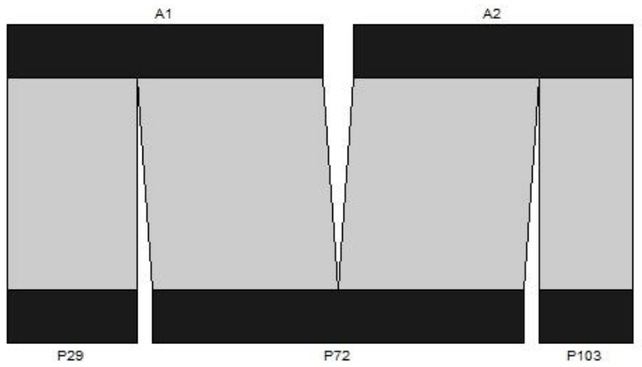

February 2018

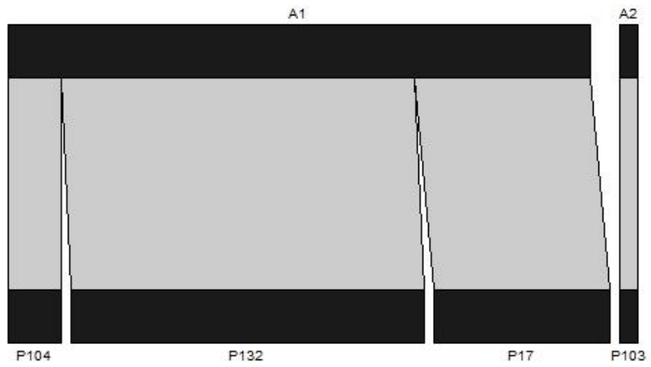

March 2018

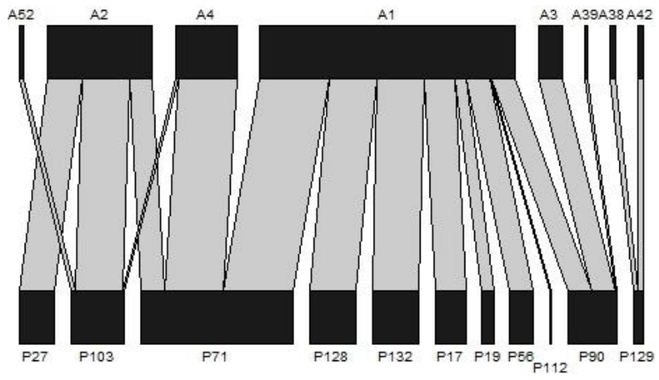

April 2018

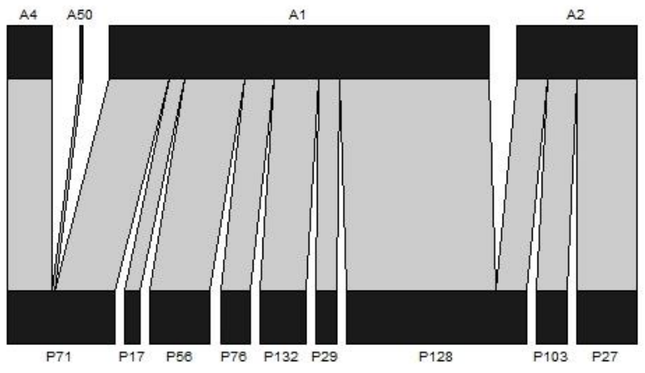

May 2018

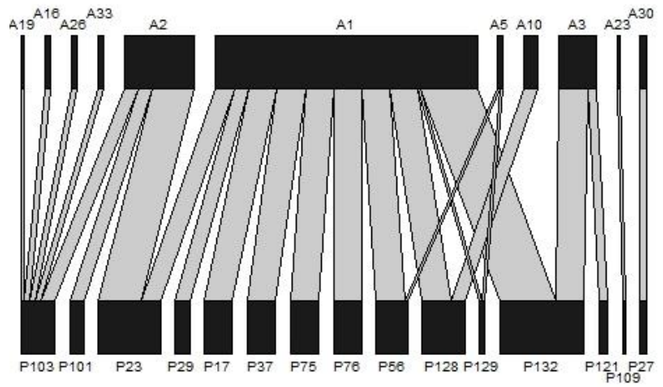

June 2018

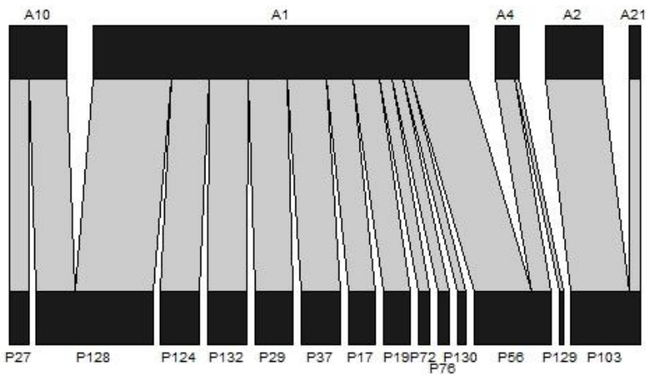

July 2018

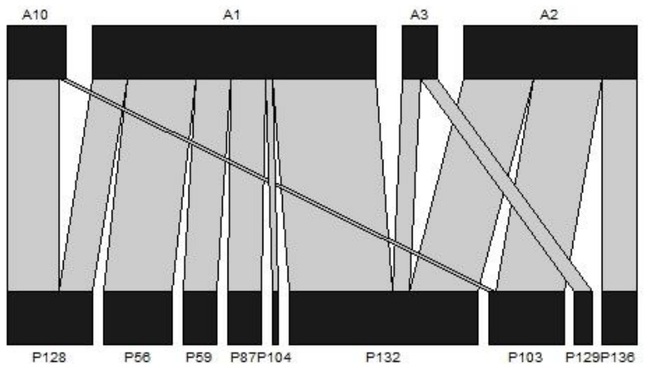

August 2018

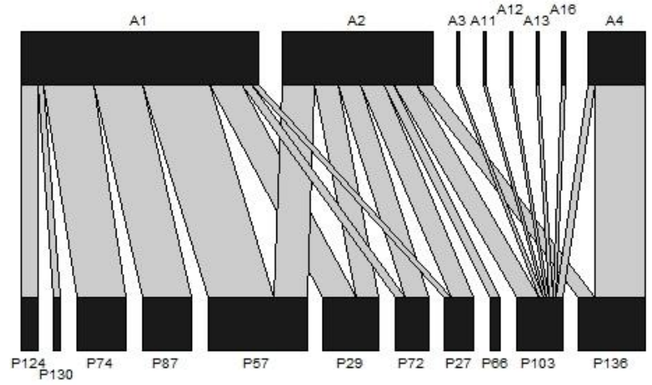

September 2018

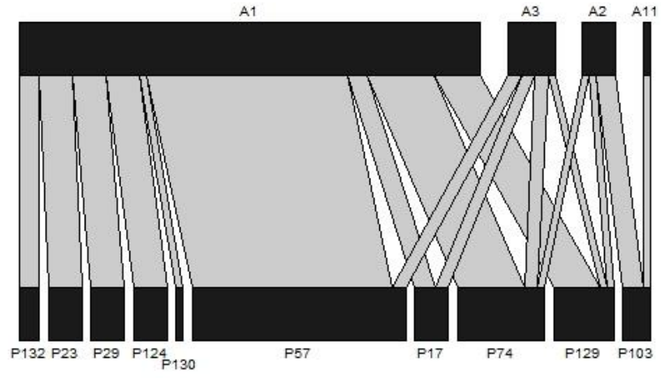

October 2018

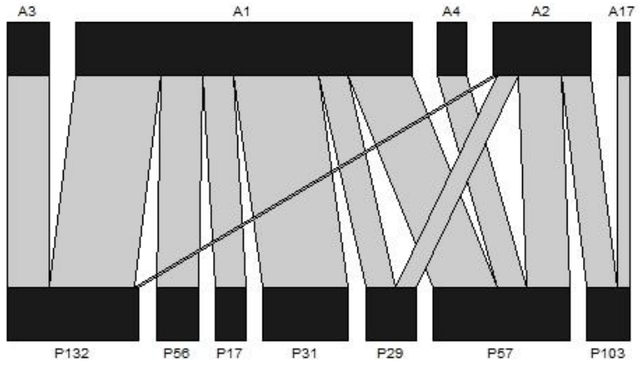

November 2018

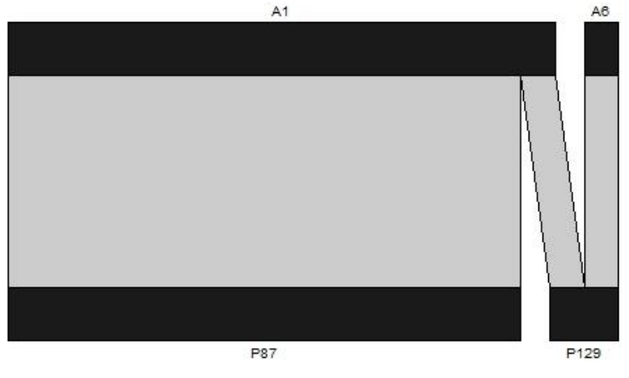

Supplement: S2 Fig — (PDF) [file pone.0230490.s002.pdf]
