# Supplementary material for: Year-round temporal stability of a tropical, urban plant-pollinator network
Source: PLoS One. 2020 Apr 10;15(4):e0230490. doi: 10.1371/journal.pone.0230490 (PMC7147774; doi:10.1371/journal.pone.0230490)
Supplement: S1 Text — (PDF) [file pone.0230490.s003.pdf]

S1 Text. Descriptions of plant-pollinator network properties examined.

Network level

connectance – the realized proportion of all possible links (number of observed links divided by the total number of possible links)

weighted connectance – linkage density divided by the number of species in the network

links per species – mean number of links per species (number of links divided by number of species)

number of compartments – the number of unconnected sections of the network (compartments are sub-sets of the web which are not connected to other compartments via either plants or pollinators)

Shannon's diversity – Shannon's diversity index of interactions; Shannon's diversity is calculated as $-\sum p_i \ln p_i$, where p_i is the proportion of *i* interactions

Group level

number of plant species – total number of plant species

number of pollinator species – total number of pollinator species

mean number of links for plants – average number of links per plant species (number of links divided by number of plant species)

mean number of links for pollinators – average number of links per pollinator species (number of links divided by number of pollinator species)

niche overlap among plants – Mean similarity in interaction pattern between plant species, calculated by Horn's index. (Values near 0 indicate no common use of niches, 1 indicates perfect niche overlap.)

niche overlap among pollinators – Mean similarity in interaction pattern between pollinator species, calculated by Horn's index. (Values near 0 indicate no common use of niches, 1 indicates perfect niche overlap.)

Species level

normalized degree (averaged across all species) - Sum of links per species, scaled by the number of possible partners

plant paired differences index (averaged across all plant species) – A measure of specialization averaged across all plant species, where 0 indicates a perfect generalist and 1 indicates a perfect specialist. This index is calculated as $\sum(P1 - Pi)/(H - 1)$, where *P1* is the highest number

of interactions in a link, *Pi* are the remaining values, and *H* is the number of potential interactors.

pollinator paired differences index (averaged across all pollinator species) – A measure of specialization averaged across all pollinator species, where 0 indicates a perfect generalist and 1 indicates a perfect specialist. This index is calculated as $\sum(P1 - Pi)/(H - 1)$, where *P1* is the highest number of interactions in a link, *Pi* are the remaining values, and *H* is the number of potential interactors.

References

Dormann CF, Gruber B, Fründ J. Introducing the bipartite package: analysing ecological networks. interaction. 2008;1(0.2413793).

Dormann CF, Fründ J, Blüthgen N, Gruber B. Indices, graphs and null models: analyzing bipartite ecological networks. The Open Ecology Journal. 2009 Feb 13;2(1).
