# Supplementary material for: Year-round temporal stability of a tropical, urban plant-pollinator network
Source: PLoS One. 2020 Apr 10;15(4):e0230490. doi: 10.1371/journal.pone.0230490 (PMC7147774; doi:10.1371/journal.pone.0230490)
Supplement: S1 Table — (PDF) [file pone.0230490.s004.pdf]

S1 Table. Pollinator species observed in Bangkok, Thailand between December 2017 – November 2018.

	Order	Family	Species	# Individuals	%	Code Name
1	Hymenoptera	Apidae	*Amegilla* sp.	27	0.34	A6
2			*Apis cerana*	555	6.89	A4
3			*Apis dorsata*	396	4.92	A3
4			*Apis florea*	1,417	17.60	A2
5			*Tetragonula* sp.	4,844	60.15	A1
6			*Xylocopa aestuans*	27	0.34	A5
7		Halictidae	*Lasioglossum* sp.	300	3.73	A10
8			*Nomia* sp.	1	0.01	A9
9		Megachilidae	*Megachile disjuncta*	7	0.09	A7
10			*Megachile sculpturalis*	3	0.04	A8
11		Scoliidae	*Campsomeris* sp.	53	0.66	A11
12			*Megacampsomeris* sp.	3	0.04	A12
13			Scoliidae 1	16	0.20	A17
14			Scoliidae 2	3	0.04	A18
15			Scoliidae 3	2	0.02	A19
16			Scoliidae 4	3	0.04	A20
17			Scoliidae 5	3	0.04	A21
18		Vespidae	*Delta pyriforme*	1	0.01	A13
19			*Polistes* sp.	4	0.05	A14
20			*Rhynchium* sp.	3	0.04	A16
21			*Ropalidia* sp.	1	0.01	A15
22			*Vespa affinis*	1	0.01	A22
23		Unknown	Unknown sp.	1	0.01	A23
24	Lepidoptera	Hesperiidae	*Suastus gremius*	1	0.01	A46
25			*Udaspes folus*	1	0.01	A43
26		Lycaenidae	*Chilades lajus*	14	0.17	A31
27			*Chilades pandava*	3	0.04	A30
28			*Leptotes plinius*	7	0.09	A42
-			*Zizina* OR *Zizula*	10	0.12	A47
29			*Zizina otis*	12	0.15	A48
30			*Zizula hylax*	7	0.09	A49
31		Nymphalidae	*Cethosia cyane*	1	0.01	A29
32			*Danaus chrysippus*	25	0.31	A33
33			*Danaus genutia*	1	0.01	A34
34			*Elymnias hypermnestra*	4	0.05	A36
35			*Euthalia aconthea*	3	0.04	A37
36			*Hypolimnas bolina*	3	0.04	A40
37			*Junonia lemonias*	1	0.01	A41
38			*Parantica agleoides*	1	0.01	A45

	Order	Family	Species	# Individuals	%	Code Name
39		Papilionidae	*Graphium doson*	3	0.04	A39
40			*Papilio demoleus*	10	0.12	A44
41		Pieridae	*Appias olferna*	4	0.05	A24
42			*Catopsilia pomona*	16	0.20	A26
43			*Catopsilia pyranthe*	1	0.01	A27
44			*Delias hyparete*	7	0.09	A35
45			*Eurema hecabe*	7	0.09	A38
46		Sphingidae	*Cephonodes hylas*	1	0.01	A28
47	Diptera	Sarcophagidae	Sarcophagidae sp.	2	0.02	A53
48		Syrphidae	*Eristalinus*	2	0.02	A52
49			Syrphidae 1	3	0.04	A54
50			Syrphidae 2	1	0.01	A55
51			Syrphidae 3	1	0.01	A56
52			Syrphidae 4	1	0.01	A57
53		Tachinidae	Tachinidae sp.	2	0.02	A58
54		Tephritidae	*Bactrocera* 1	16	0.20	A50
55			*Bactrocera* 2	1	0.01	A51
56			Tephritidae sp.	165	2.05	A59
57		Unknown	Unknown sp.	5	0.06	A60
58	Hemiptera	Rhopalidae	Rhopalidae sp.	40	0.50	A61
Total				8,053	100	

Code names refer to the labels used in the pollination networks (S2 Fig).
