# Supplementary material for: Year-round temporal stability of a tropical, urban plant-pollinator network
Source: PLoS One. 2020 Apr 10;15(4):e0230490. doi: 10.1371/journal.pone.0230490 (PMC7147774; doi:10.1371/journal.pone.0230490)
Supplement: S2 Table — (PDF) [file pone.0230490.s005.pdf]

S2 Table. Plant species at which pollinator observations were conducted (Bangkok, Thailand; December 2017 – November 2018).

	Family	Species	Status	Code Name
1	Acanthaceae	*Andrographis paniculata* (Burm.f.) Nees	unclear	P9
2		*Asystasia gangetica* (L.) T.Anders.	native	P12
3		*Crossandra infundibuliformis* (L.) Nees	native	P42
4		*Justicia betonica* L.	exotic	P71
5		*Justicia brandegeeana* Wassh. & L.B.Sm.	exotic	P72
6		*Pseuderanthemum curtatum* (C. B. Cl.) Merrill	unclear	P99
7		*Pseuderanthemum carruthersii* (Seem.) Guill.	exotic	P100
8		*Ruellia simplex* C.Wright	exotic	P104
9		*Ruspolia hypocrateriformis* (Vahl) Milne-Redh.	exotic	P105
10		*Thunbergia grandiflora* (Roxb. ex Rottl.) Roxb.	unclear	P125
11	Alismataceae	*Echinodorus cordifolius* (L.) Griseb	exotic	P49
12	Amaranthaceae	*Amaranthus viridis* L.	exotic	P8
13		*Celosia argentea* L.	native	P27
14		*Gomphrena globosa* L.	native	P57
15	Amaryllidaceae	*Crinum asiaticum* L.	native	P41
16		*Hymenocallis littoralis* (Jacq.) Salisb.	exotic	P64
17	Anacardiaceae	*Mangifera indica* L.	native	P79
18	Annonaceae	*Cananga odorata* (Lam.) Hook.f. & Thomson	exotic	P21
19	Apocynaceae	*Adenium obesum* (Forssk.) Roem. & Schult.	exotic	P2
20		*Allamanda blanchetii* A.DC.	exotic	P4
21		*Allamanda cathartica* L.	exotic	P5
22		*Alstonia scholaris* (L.) R. Br.	native	P7
23		*Catharanthus roseus* (L.) G.Don	exotic	P26
24		*Cerbera odollam* Gaertn.	native	P28
25		*Kopsia arborea* Blume	native	P73
26		*Nerium oleander* L.	exotic	P85
27		*Pentalinon luteum* (L.) B.F.Hansen & Wunderlin	exotic	P92
28		*Plumeria* spp.	exotic	P96
29		*Tabernaemontana divaricata* (L.) R.Br. ex Roem. & Schult.	native	P118
30		*Wrightia religiosa* (Teijsm. & Binn.) Benth. ex Kurz	native	P131
31	Araceae	*Spathiphyllum cannifolium* (Dryand. ex Sims) Schott	exotic	P115
32	Arecaceae	*Ptychosperma macarthurii* (H.Wendl. ex H.J.Veitch) H.Wendl. ex Hook.f.	exotic	P101
33	Balsaminaceae	*Impatiens balsamina* L.	exotic	P66
34		*Impatiens* sp.	--	P65
35	Bignoniaceae	*Bignonia magnifica* W.Bull	exotic	P109
36		*Crescentia alata* Kunth	exotic	P40
37		*Tabebuia aurea* (Silva Manso) Benth. & Hook.f. ex S.Moore	exotic	P116
38		*Tabebuia rosea* (Bertol.) Bertero ex A.DC.	exotic	P117
39		*Tecoma stans* (L.) Juss. ex Kunth	exotic	P121

	Family	Species	Status	Code Name
40	Boraginaceae	*Cordia sebestena* L.	exotic	P37
41		*Ehretia monopyrena* Gottschling & Hilger	native	P23
42		*Heliotropium indicum* L.	native	P61
43	Cannaceae	*Canna indica* L.	exotic	P22
44	Cleomaceae	*Tarenaya spinosa* (Jacq.) Rafin.	exotic	P31
45	Combretaceae	*Combretum erythrophyllum* (Burch.) Sond.	exotic	P34
46		*Combretum indicum* (L.) C. C. H. Jongkind	native	P35
47	Compositaea	*Chrysanthemum morifolium* (Ramat.)	exotic	P45
48		*Cosmos* sp.	exotic	P38
49		*Cosmos sulphureus* Cav.	exotic	P39
50		*Eclipta prostrata* (L.) L.	unclear	P50
51		*Helianthus annuus* L.	exotic	P59
52		*Rudbeckia laciniata* L.	exotic	P103
53		*Sphagneticola trilobata* (L.) Pruski	exotic	P130
54		*Tagetes erecta* L.	exotic	P119
55		*Thymophylla tenuiloba* (DC.) Small	exotic	P126
56		*Tithonia rotundifolia* (Mill.) S.F.Blake	exotic	P127
57		*Zinnia angustifolia* Kunth	exotic	P135
58		*Zinnia violacea* Cav.	exotic	P136
59	Convolvaceae	*Convolvulus sabatius* Viv.	exotic	P36
60		*Ipomoea carnea* Jacq.	exotic	P67
61	Costaceae	*Hellenia speciosa* (J.Koenig) S.R.Dutta	native	P29
62	Dilleniaceae	*Tetracera loureiri* (Finet & Gagnep.) Pierre ex W. G. Craib	native	P122
63	Dipterocarpaceae	*Shorea robusta* Gaertn.	unclear	P111
64	Euphorbiaceae	*Euphorbia milii* Des Moul.	exotic	P53
65		*Jatropha integerrima* Jacq.	exotic	P70
66	Heliconiaceae	*Heliconia* spp.	exotic	P60
67	Lamiaceae	*Callicarpa americana* L.	exotic	P19
68		*Coleus scutellarioides* (L.) Benth.	native	P94
69		*Gmelina philippensis* Cham.	native	P56
70		*Ocimum basilicum* L.	native	P87
71		*Orthosiphon aristatus* (Blume) Miq.	native	P88
72		*Rotheca microphylla* (Blume) Callm. & Phillipson	exotic	P32
73		*Salvia divinorum* Epling & Játiva	exotic	P107
74	Leguminosae	*Bauhinia acuminata* L.	native	P13
75		*Bauhinia purpurea* L.	native	P14
76		*Bauhinia tomentosa* L.	native	P15
77		*Caesalpinia pulcherrima* (L.) Sw.	exotic	P17
78		*Calliandra haematocephala* Hassk.	unclear	P18
79		*Cassia bakeriana* Craib	native	P24
80		*Cassia fistula* L.	native	P25
81		*Clitoria ternatea* L.	native	P33
82		*Crotalaria juncea* L.	native	P43

	Family	Species	Status	Code Name
83	Leguminosae	*Erythrina variegata* L.	native	P51
84		*Peltophorum pterocarpum* (DC.) K.Heyne	native	P91
85		*Saraca indica* L.	native	P108
86		*Senna siamea* (Lam.) H.S.Irwin & Barneby	native	P110
87	Linderniaceae	*Torenia fournieri* Linden ex E. Fourn.	native	P128
88	Lythraceae	*Cuphea hyssopifolia* Kunth	exotic	P44
89		*Lagerstroemia floribunda* Jack	native	P74
90		*Lagerstroemia indica* L.	native	P75
91		*Lagerstroemia speciosa* (L.) Pers.	native	P76
92	Malpighiaceae	*Galphimia glauca* Cav.	exotic	P124
93	Malvaceae	*Abelmoschus moschatus* (L.) Medicus	unclear	P1
94		*Alcea rosea* L.	exotic	P3
95		*Dombeya* sp.	--	P47
96		*Hibiscus schizopetalus* (Dyer) Hook.f.	exotic	P62
97		*Hibiscus syriacus* L.	exotic	P63
98	Marantaceae	*Thalia geniculata* L.	exotic	P123
99	Musaceae	*Musa acuminata* Colla	native	P83
100	Myrtaceae	*Callistemon lanceolatus (Sm.) Sweet*	exotic	P20
101		*Xanthostemon chrysanthus* (F.Muell.) Benth.	exotic	P132
102		*Xanthostemon verdugonianus* Náves ex Fern.-Vill.	exotic	P133
103	Nyctaginaceae	*Bougainvillea* hybrid	exotic	P16
104	Nymphaeaceae	*Nymphaea* sp.	--	P86
105	Oleaceae	*Jasminum* sp.	--	P69
106	Orchidaceae	*Dendrobium* sp.	--	P46
107	Passifloraceae	*Turnera ulmifolia* L.	exotic	P129
108	Plantaginaceae	*Achetaria azurea* (Linden) V.C.Souza	exotic	P89
109		*Angelonia goyazensis* G. Benth.	exotic	P10
110		*Russelia equisetiformis* Schltdl. & Cham.	exotic	P106
111	Plumbaginaceae	*Plumbago auriculata* Lam.	exotic	P95
112	Poaceae	unknown sp.	--	P58
113		*Zea mays* L.	exotic	P134
114	Portulaceae	*Portulaca grandiflora* Hook.	exotic	P97
115		*Portulaca oleracea* L.	unclear	P98
116	Rosaceae	*Rosa* spp.	--	P102
117	Rubiaceae	*Arachnothryx leucophylla* (Kunth) Planch.	exotic	P11
118		*Gardenia carinata* Wall. ex Roxb.	native	P54
119		*Gardenia* spp.	--	P55
120		*Ixora lobbii* Loudon	native	P68
121		*Morinda citrifolia* L.	native	P81
122		*Mussaenda philippica* A.Rich.	native	P84
123		*Oxyceros horridus* Lour.	native	P90
124		*Tarenna wallichii* (Hook.f.) Ridl.	native	P120
125	Rutaceae	*Murraya paniculata* (L.) Jack	native	P82

	Family	Species	Status	Code Name
126	Sapotaceae	*Mimusops elengi* L.	native	P80
127	Scrophulariaceae	*Leucophyllum frutescens* (Berland.) I.M. Johnst.	exotic	P78
128	Solanaceae	*Petunia atkinsiana* D. Don ex Loud.	exotic	P93
129		*Solandra grandiflora* Sw.	exotic	P112
130		*Solanum torvum* Sw.	exotic	P113
131		*Solanum virginianum* L.	native	P114
132	Verbenaceae	*Citharexylum spinosum* L.	exotic	P30
133		*Duranta erecta* L.	exotic	P48
134		*Lantana camara* L.	exotic	P77
135	Zingiberaceae	*Alpinia galanga* (L.) Willd.	native	P6
136		*Etlingera elatior* (Jack) R.M.Sm.	native	P52

Status information was obtained from the Catalogue of Life website (http://www.catalogueoflife.org; accessed November 2019): native – listed as native in Thailand or Southeast Asia; exotic – listed as introduced in Thailand or Southeast Asia; unclear – distribution data is not clear (e.g., listed as native in certain neighboring countries, but no information reported for Thailand). Code names refer to the labels used in the pollination networks (S2 Fig).
