# Supplementary material for: Year-round temporal stability of a tropical, urban plant-pollinator network
Source: PLoS One. 2020 Apr 10;15(4):e0230490. doi: 10.1371/journal.pone.0230490 (PMC7147774; doi:10.1371/journal.pone.0230490)
Supplement: Attachment — Submitted filename: Response to Reviewers.docx [file pone.0230490.s006.docx]

We greatly appreciate the thoughtful and constructive comments provided by both reviewers, and believe our revised manuscript has been substantially strengthened. Please see our detailed responses to each comment below. (Red = new text added to the manuscript)

**Reviewer #1**: The study titled “Year-round temporal stability of a tropical, urban pollination network” is interesting and a result well-worthy of publication. The presentation is concise and brings out the important conclusions convincingly. However, there are a few problems that should be addressed for clarity and so that the readers fully understand the implications of the results.

1. One is a fuller description of the parks. Parks can range from relatively “wild” areas that rely on natural precipitation to highly manicured gardens that are regularly watered. As I read the paper, it became increasing evident that the parks in this study are highly manicured (I think this is correct). This should be conveyed in the introduction and stated again in the methods. If the authors could document how many of the plant species they sampled were native or domesticated, that would give the readers an idea of the types of parks sampled in the study. Watering the plants surely decreased the variation in floral abundance and is partly to explain why the pollination networks change so little through the year.

We have added a fuller description of the parks in both the intro and the methods, as suggested.

Intro: “…Finally, the abundance of floral resources found year-round in tropical cities [29], often due to the mix of native and exotic plant species found in cultivated parks, provides a unique opportunity to study plant-pollinator interactions in a relatively stable environment. Therefore, the objectives of this study were to examine plant-pollinator interactions at public parks in Bangkok, Thailand across 12 consecutive months, and to assess the stability of network properties throughout this period.”

Methods: “All nine parks were regularly watered, but experienced different degrees of floral rotation; some parks periodically brought in new flowering plant species as previously planted species dropped their flowers, while other parks did not rotate plant species at all (A. Stewart, pers. obs.). It is common for parks in Bangkok to have both native and exotic plant species [30].”

We have also included status info (native or exotic) for each studied plant species in S2 Table, and included a sentence in the Discussion: “Urban parks often favor plant species with showy flowers, and cultivate a mix of native and exotic species that provide abundant floral resources year-round [29,36,37]. Indeed, of the 136 plant taxa observed in this study, 73 species were exotic and 47 were native; the remaining 16 could not be verified (S2 Table).”

2. Second, the title states that the study is about temporal changes over the year, but the comparisons of floral abundance, floral species richness, etc. are conclusions that are based on differences across parks. If this is the case, the study is also about spatial changes. This should be addressed.

Yes, this study was conducted specifically to examine temporal changes. Data was collected from multiple parks in order to provide replication for the study, but we were not interested in comparing spatial differences across parks (which we already did in Stewart et al. 2018). This is why we included park as a random factor in our analyses (as stated in the Methods). Our results show that, even when taking into account the fact that some parks have more floral resources on average than others, floral abundance and richness still significantly influence pollinator abundance and richness.

3. Finally, and importantly, is choice of plant species sampled at each park. The text is vague on this and needs to provide more detail. Line 80 states that the plants where observations were made were those that were “the most abundant plant species that were in flower”. How was this determined? How many plants were studied at each park and each date? Why did the study use this approach and not a protocol that sampled the same plant species no matter how many flowers each species had? It is important that the protocol used is justified.

Both reviewers expressed confusion about our sampling methods. We attempted to clarify this under the “Data collection” section in the Methods:

“During each sampling event, we conducted 15-minute observations at all of the most abundant plant species that were in flower. In order to be efficient with sampling, we only observed plant species with at least 20 flowers or inflorescences; this resulted in approximately 80 – 90% of the flowering plant community being sampled at each park in each month. We chose this sampling method because we wanted to maximize the number of flowering plant species sampled in order to track floral resource availability across months, and to record as many pollination interactions as possible, but were limited by manpower.”

4. This protocol has important implications for what the measures of floral abundance mean. As written it appears that your **2 x 2m plots is extrapolated to the size of the entire park which would result in a gross overestimate of actual floral abundance**. Please explain all this more carefully and discuss how this choice influences the interpretation of your results, or it does at all.

Sorry for the confusion, we have tried to clarify:

“Floral abundance was determined at each plot as the number of flowers per 2 x 2 m plot; these values were then averaged to obtain a mean number of flowers per plot for each park in each month. Floral richness was determined as the number of flowering plant species sampled at each park in each month.”

5. L. 22,23- this relates to one of my larger comments above. This study is about tropical pollination networks that are probably more stable than networks in the temperate zone, but also the study is in highly manage parks. That should be made clear at this point.

Revised as suggested:

Abstract: “… tropical urban parks provide a unique opportunity to study network stability in an environment where temperature and floral resources are relatively constant due to both the tropical climate as well as park horticulture.”

6. L. 29. Add that when “all parks are pooled” there is no variation in pollinator abundance or richness.

Revised as suggested: “We found that neither pollinator abundance nor richness varied significantly across months when all parks were pooled.”

7. L. 30-Add that when parks are compared, there is evidence that floral abundance, etc. influence pollinator richness.

We do state these results in the abstract: “However, pollinator abundance was significantly influenced by floral abundance, floral richness, and their interaction, and pollinator richness was significantly influenced by floral richness and precipitation.”

(We did not compare parks for these analyses, park was included as a random factor as explained above.)

8. L.80. How did you determine when plants were abundant or rare?

This has now been clarified (see response to comment 3).

9. L. 86 swing could be deleted

The word “swing” was removed as suggested.

10. L 93-95. Please be more explicit and complete about how floral abundance and richness are determined, and extrapolated to total park size, etc. How did you minimize bias?

This has now been clarified (see response to comment 4).

11. L109. As stated above, you examined how pollinator communities varied over time (month to month comparisons when all parks are polled), and space (given that the number of flowering plants and floral abundance were compared across parks.

We did not compare across parks (see response to comment 2).

12. L. 112. The use of the term “trophic level” is confusing given that there is only plants and their pollinators. Should this be compartments, or some other term?

The term “trophic level” is commonly used in pollination networks to distinguish between plants and pollinators (Dormann et al. 2009). (The term “compartments” cannot be used here because this term actually has a different meaning in bipartite networks: subsets of the network that are not connected to other subsets.)

13. L. 198-199-vaguely written. Rewrite for clarity.

We have revised this sentence from…

“This justification is also consistent with our results, since flowers were abundant year-round in our study parks, thus maintaining high pollinator abundance across seasons.”

… to the following:

“This justification is also consistent with our results; if insect abundance is primarily driven by food abundance, it seems logical that the year-round supply of flowers in our study parks are able to maintain high pollinator abundance across seasons.”

14. L 225. Change “within” to “of”

Changed as suggested.

15. L 250. either delete or rewrite the phrase “…that are undeterred by human activity.”

We have revised this sentence: “However, it is important to note that these parks are likely only suitable for pollinator species with generalist foraging and nesting habits, and that are tolerant of human activity.

16. L270. This reference is improperly formatted

Thank you for catching our mistake.

17. Add scale bar to map of parks

The map already has a scale bar, but we have made it bigger so that it is more noticeable.

**Reviewer #2**: The presented results give an important and new insight into pollinator communities of tropical cities and I think they should be published. However, data collection had some limitations, limiting also the interpretation of data which should be outlined a little more clearly.

1. First of all, when recording insect visitation to plants, you do not have information on whether the insects were pollinating the plants (even when the insect was observed to touch the reproductive parts of the flower). Therefore, I would caution against using the term pollination network. Instead, I would suggest to rather use the term plant insect/pollinator visitation network.

“Pollination network” is the standard term that is used for this type of study, even if there is no concrete evidence whether the animals are true pollinators or not. Indeed, most other studies label ALL floral visitors as “pollinators” (see Olesen et al. 2008, Petanidou et al. 2008, Dupont et al. 2009, Goldstein & Zych 2016, Robinson et al. 2018) or use the same criteria that we used (contact with floral reproductive structures; see Alarcón et al. 2008, Pauw & Stanway 2015, CaraDonna et al. 2017, Sritongchuay et al. 2019). In order to be consistent with the terminology used by these other studies, we prefer to continue using the term “pollination network”.

2. Furthermore, only the most abundant plants in the parks were observed which means that only a fraction of the entire network was selected which also imposes a strong bias for the assessment of network properties. The use of this method also means that the habitat variables floral abundance and floral richness are actually not representative for the park which could easily be misunderstood by the reader. Floral abundance was based on 2x2 m plots with particularly abundant plants. And floral richness included only those most abundant plants. While these parameters can still be related to observed plant visitors, it should be pointed out more clearly for the interpretation of the results.

Both reviewers expressed confusion about our sampling methods. We attempted to clarify this, please see response to comment 3 from Reviewer 1.

3. Additionally, the analyses with linear mixed modelling should be revised and explained more detailed. **(a)** Your description is a little unclear, but it sounds like you calculated several independent models for each climatic and habitat variable which, in my opinion, is inadequate. Including month as a random factor in some models, although it was not significant as a fixed factor in another model for the same response variable illustrates part of the problem. All climatic and habitat variables that could potentially affect pollinator abundance or richness should be examined in one model (per response variable). **(b)** You should also consider using the same explanatory variables (as used for pollinator abundance and richness) for the analyses of the network properties. **(c)** Using the linear mixed model method (LMM) requires normal distribution of the data. Therefore, you should state whether data was normally distributed. **(d)** The results of the LMM should also include R2-values. **(e)** The graphs of model predictions (Fig. 2) show always three curves, for low, medium and high predictions (Fig. 2, A-C), except for one of the graphs (Fig. 2, D). It is not explained what these predictions are based on and why Fig. 2, D has only one curve. Typically, there would be just one curve per response variable and explanatory variable. Please either provide additional information or change the graphs.
(a) Thank you for the advice. We re-ran the analyses so that all climatic and habitat variables were examined in the same model. We have revised the methods accordingly: “We used linear mixed modelling (LMM; package “lme4”) to examine which predictors influenced pollinator abundance and richness. The response variables examined were total pollinator abundance, total pollinator richness, and the abundance and richness of the most common insect orders (Hymenoptera, Lepidoptera, and Diptera). The fixed factors tested were month, temperature, precipitation, temperature x precipitation, floral abundance, floral richness, and floral abundance x floral richness; park was included as a random factor.” Re-running the analyses did not change most of our results, but there were slight differences for Hymenopteran richness, Lepidopteran abundance, and Lepidopteran richness, so we revised the Results (2^nd^ paragraph of Results, Table 1, and Figure 2) and Discussion (5^th^ paragraph of the Discussion) accordingly.
(b) For network properties, we were primarily interested in whether the networks changed across months, or were stable over time, which is why month was the only fixed factor.
(c) We have now included this info in the methods: “QQ plots (package “stats”) were used to check the normality of the residuals.”
(d) We have now included this info in Table 1.
(e) The multiple curves allow us to illustrate the effect of two significant predictors, with one predictor along the x-axis and the other predictor denoted by color. We have revised the figure legend to try to make this more clear: “(A) Total pollinator abundance was significantly influenced by floral abundance (x-axis), floral richness (denoted by color; red: 6.6 spp., blue: 13.9 spp., green: 21.1 spp.), and their interaction. (B) Total pollinator richness was significantly influenced by floral richness (x-axis) and precipitation (denoted by color; red: 58 mm, blue: 155 mm, green: 251 mm). (C) Hymenopteran abundance was significantly influenced by floral abundance (x-axis), floral richness (denoted by color; red: 6.6 spp., blue: 13.9 spp., green: 21.1 spp.), and their interaction. (D) Hymenopteran richness was significantly influenced by floral richness (x-axis) and precipitation (denoted by color; red: 58 mm, blue: 155 mm, green: 251 mm). (E) Lepidopteran abundance and (F) Lepidopteran richness were significantly influenced by floral richness (x-axis).”
4. Another critical point is the evaluated effect of precipitation. The significance of precipitation as a predictor for any response variable is questionable, because all parks were regularly watered additionally to natural precipitation which the model does not take into account. This influence should be considered in analyses and interpretation.

While it is unlikely that precipitation influenced the plants, it may have influenced other aspects of the insects’ life history and/or behavior. We have clarified this in the Discussion: “Precipitation likely had little effect on park vegetation and floral resources, given regular watering and park horticulture, but it possibly influenced other aspects of insect life history and/or behavior. Findings from previous studies are mixed. For example, …”

5. Finally, you state in your conclusion that the studied urban tropical environments host abundant and diverse bee communities. However, you do not relate pollinator abundance or species richness to pollinator communities of natural habitats within the same region. If possible, this information should be added. Otherwise, the value of these urban habitats remains unclear.

Good point. There is not much published on pollinator diversity in Thailand, but we have added one reference to the Conclusion: “For example, we only observed 58 insect taxa, in contrast to a recent study in southern Thailand that recorded 316 insect pollinator taxa [53].”

6. Overall, the manuscript is very nicely written and the clear language makes it easy to follow. I hope that you find my comments helpful for revising your manuscript. I think it will be an interesting and valuable addition to the literature.

Thank you! We greatly appreciate your thoughtful and constructive comments.

Minor comments

Abstract

7. L32 You mention a change of species composition here in the abstract, but do not include this information in the result section. Please add this in the results.

True. We have added the following information.

Methods: “We also calculated species turnover (proportion of species lost or gained between two consecutive months) for both plants and pollinators using the “codyn” package [35].”

Results: “We did, however, find that both plant and pollinator composition changed across months; species turnover between consecutive months was close to 50% for both plants (mean ± SE: 0.45 ± 0.04) and pollinators (0.46 ± 0.04).”

Introduction

8. L40 Consider adding another more general reference additional to 1 & 2.

Reference added as suggested: “Faegri K, van der Pijl L. The principles of pollination ecology. 2nd ed. Pergamon Press Ltd.; 1971.”

Methods

9. L71 Maybe number of inhabitants and area of Bangkok could be added.

We have added this info: “Bangkok is the most populated city in Thailand (over 9.6 million residents according to the 2010 census)…”

10. L73-75 What was the size of the parks?

We have added this info to S1 Figure. “We collected data from 9 parks spaced throughout the city (S1 Fig). These nine parks were selected because they are all at least 1 km apart, and represent a diversity of park sizes (S1 Fig)…”

11. L79-81 What was the range of number of plant species observed per park or in other words how many different plots did you observe per park? Were plots fixed or newly assigned for each observation?

We have added this info to the Methods: “Plots were not fixed; they varied by month according to which plant species were flowering (mean ± SE plant species sampled per park per month: 13.8 ± 0.7 species; range: 1-33 species).”

12. L87 Within how many days did you observe all nine parks each month? Was the order always the same?

We have added this info to the Methods: “The order in which parks were visited during each month was random, and sampling was only conducted on sunny days.”

13. L90 “average” instead of “averagely”

Thanks for catching that typo.

14. L90-93 As you did not measure temperature yourself, I would rephrase: We also obtained data on… from …

Revised as suggested.

15. L98-116 Please revise your approach of LMM and add details as suggested above.

Revised as suggested (see response to comment 3).

Results

16. L130-133 Please explain what points and bars represent.

Thanks for catching this oversight. We have added this info (“points and error bars represent mean ± SE”).

17. L135-142 Please indicate whether the listed significant explanatory variables increase or decrease the response variables.

We added this info as suggested:

When examining total pollinator abundance, we found that neither month, temperature, precipitation, nor temperature x precipitation had a significant effect, but both floral abundance and floral richness had positive effects, and their interaction was significant as well (Table 1; Fig 2A). For total pollinator richness, both precipitation and floral richness had significant positive effects (Table 1; Fig 2B). Hymenoptera abundance was significantly influenced by floral abundance (positive effect), floral richness (positive effect), and their interaction (Table1; Fig 2C), while Hymenoptera richness was influenced by precipitation and floral richness (positive effects; Table 1; Fig 2D). Both Lepidoptera abundance and richness were positively influenced by floral richness (Table 1; Fig 2E-F).”

18. L145-149 Consider making significant predictors bold for easier reading in black and white.

Revised as suggested.

19. L151-157 Please explain what low, medium and high predictions are based on or change as suggested above.

We have added this info to Figure 2. “(A) Total pollinator abundance was significantly influenced by floral abundance (x-axis), floral richness (denoted by color; red: 6.6 spp., blue: 13.9 spp., green: 21.1 spp.), and their interaction. (B) Total pollinator richness was significantly influenced by floral richness (x-axis) and precipitation (denoted by color; red: 58 mm, blue: 155 mm, green: 251 mm). (C) Hymenopteran abundance was significantly influenced by floral abundance (x-axis), floral richness (denoted by color; red: 6.6 spp., blue: 13.9 spp., green: 21.1 spp.), and their interaction. (D) Hymenopteran richness was significantly influenced by floral richness (x-axis) and precipitation (denoted by color; red: 58 mm, blue: 155 mm, green: 251 mm). (E) Lepidopteran abundance and (F) Lepidopteran richness were significantly influenced by floral richness (x-axis).”

20. L154-156 Graph B does not show precipitation as suggested by the caption. Likewise, graph C does not show floral richness nor the interaction of floral abundance and richness.

I think perhaps the reviewer missed this information in the caption. To make this more clear, we have included more precise info to the figure caption (see response to comment 3e and comment 19).

Discussion

21. L195 …most orders of insects…

Thank you for catching that typo.

Conclusion

22. L245-246 Without a comparison to pollinator communities of natural or other kind of habitats in the same region, it is not really possible to make a statement on whether the recorded pollinator community is very abundant or species rich or how valuable of a habitat the parks are.

We have addressed this point, please see our response to comment 5.
