# Supplementary material for: Year-round temporal stability of a tropical, urban plant-pollinator network
Source: PLoS One. 2020 Apr 10;15(4):e0230490. doi: 10.1371/journal.pone.0230490 (PMC7147774; doi:10.1371/journal.pone.0230490)
Supplement: Attachment — Submitted filename: Response to Reviewers.docx [file pone.0230490.s007.docx]

**Response to Reviewer Comments** (second round)

(Red = new text added to the manuscript)

**Reviewer #1**: The manuscript is much clearer and more concise. I have no comments and recommend it for publication.

Thank you!

**Reviewer #2**: The revised manuscript is much improved and most concerns were addressed and changes performed accordingly. A few unclarities and concerns remain and I would suggest some more minor revisions.

Thank you for your thorough consideration of our manuscript.

Following up on 1. of my previous comments and the authors reply: Although some studies use the term pollination network, the use of this terminology is problematic and actually incorrect as research has shown that pollination cannot be inferred from flower visitation (see e.g. King et al. 2013 or Popic et al. 2013). Less problematic is the use of plant pollinator networks which I suggest as one alternative to pollination networks and which numerous other studies use (including many you have cited, i.e. Robinson et al. 2018, Alarcón et al. 2008 or CaraDonna et al. 2017). However, the most accurate term is plant insect networks (used e.g. by Vanbergen et al. 2014 or Losapio et al. 2015). For improvements in the fields of pollination ecology and plant insect interactions, I would encourage the use of plant insect networks when visitation and not pollination was assessed like in this study.

We completely understand your reasoning, and we have revised our manuscript to reflect your rationale while still being accurate to how we collected data. We realize that not all visitors are pollinators, and we were very intentional during data collection to only record visitors that contacted floral reproductive structures (as we state in the Methods). Therefore, our study does not reflect total insect visitation, but only the visitation of potential pollinators, and we have revised the manuscript text to clarify this (i.e., that our network reflects only potential pollinators, and that some of the taxa in our networks may simply be visitors that do not actually transfer pollen for certain plant species). Thank you for your suggestions to use “plant-pollinator networks” or “plant-insect networks,” which we have incorporated into the text as appropriate.

Title: “Year-round temporal stability of a tropical, urban plant-pollinator network”

Abstract:

“The objectives of this study were thus to examine the interactions between flowering plants and their potential pollinators in a large, tropical city …”

“We conclude that the year-round constancy of floral resources and climate conditions appear to create a ~~pollination~~ network in dynamic equilibrium, where plant and pollinator species compositions change, but network properties remain stable.”

Intro:

“Therefore, the objectives of this study were to examine the interactions between flowering plants and their potential pollinators at public parks in Bangkok, Thailand…”

Methods:

“… to record as many plant-pollinator interactions as possible…”

“… all animals that contacted floral reproductive structures while visiting flowers (i.e., potential pollinators) were recorded. As we did not quantify actual pollen transfer, we cannot confirm that all recorded taxa were true pollinators; it is likely that observed animal taxa differ in pollination effectiveness, and some taxa may not have contributed to pollination at all.”

Results:

“… we observed 8,053 potential pollinators…”

“The overall plant-pollinator network…”

[we have also revised the wording in Table 2, S2 Figure, and S1 Text]

Discussion:

“When examining plant-pollinator networks…”

“… the temporal stability of plant-pollinator network properties…”

“… create a ~~pollination~~ network in dynamic equilibrium, where plant and pollinator species…”

Following up on 2. of my previous comments and the authors reply: The sampling methods for determining floral abundance and richness were clarified. However, the resulting bias and limitations for the interpretation of results is still not sufficiently discussed in my opinion. The described selective sampling only from the most abundant flowers creates a bias for network analyses, because plants present in low numbers are consistently missing. Please consider this bias in the discussion. Furthermore, the variables floral abundance and floral richness are not representative for the actual park vegetation as they are based on the most abundant patches of flowers which is a selective measure. Please make this more clear in the discussion to avoid confusion.

We have clarified both points in the Discussion as suggested:

“Our networks reflect the large majority of plant species found in the parks, although our inability to collect data on the rarest plant species may have influenced some network property estimates.”

“It is important to note that our measures of pollinator abundance were obtained from our study plots of flowering plant species, and that many areas of the parks had substantially lower floral abundance; such areas would undoubtedly have fewer pollinators than was observed at our study plots.”

L89 It still remains unclear if only one park a day was observed, all in one day or how many parks per day within what time span of a month (e.g. three parks a day, and all parks within first week of the month).

All sampling at each park was conducted within a single day, and up to three parks were observed in a single day. During each month, the first and last parks were sampled within 16-25 days of each other.

L96 visitation interactions instead of pollination interaction

We have changed “pollination interactions” to “plant-pollinator interactions” following the suggestion in comment 1 above.

L112-113 If only those flowers were observed that had a minimum of 20 flowers/inflorescences this calculation does not really reflect the floral abundance of the park.

We have addressed this as suggested (see comment 2 above).

L164-167 Suggestion for consistency/easy reading: either individual brackets with “(positive effect)” always after the first and second predictor or “(positive effects)” only after the second predictor.

The reason why we can’t do this is because some sentences include significant *interactions* between predictors, which are neither positive or negative.

L180-189/Fig. 2: I find the interpretation of the graphs still a little difficult. Can you provide more information on the visualization of the model predictions in the methods section?

Information added as requested in the Methods (“Data analysis” section):

“In the model prediction graphs, when two explanatory predictors were found to be significant, one predictor was plotted along the x-axis and the second predictor was represented using various colors; this method also clearly shows when the two predictors have a significant interaction (non-parallel lines) or not (parallel lines).”

L272-278 As mentioned above, you should point out that the visitation networks analyzed completely omitted less abundant plants which is potentially affecting network properties.

Revised as suggested:

“However, it is important to note that (1) our networks included only the most abundant plant species, which may over- or underestimate some network properties, and (2) these parks are likely only suitable for pollinator species with generalist foraging and nesting habits, and that are tolerant of human activity.”

L289-290 Maybe this could be rephrased slightly, because considering the much lower species diversity compared to the below mentioned fruit orchards, the pollinator community in the parks does not appear to have a very high level of diversity.

We have revised the sentence as follows:

“The findings from this study reveal that tropical urban parks are capable of supporting stable ~~diverse and abundant~~ pollinator communities year-round.”
